# Supplementary material for: Essential angiosperm-specific subunits of HDA19 histone deacetylase complexes in Arabidopsis
Source: EMBO J. 2025 Apr 28;44(12):3521–46. doi: 10.1038/s44318-025-00445-w (PMC12170880; doi:10.1038/s44318-025-00445-w)
Supplement: Supplementary file 1 — Appendix [file 44318_2025_445_MOESM1_ESM.pdf]

Essential angiosperm-specific subunits of HDA19 histone deacetylase complexes in Arabidopsis

Appendix table of contents

Appendix Figure S1. The expression patterns of genes encoding HDIP1/2/3 and other HDAC components in Arabidopsis……………………………………..…………………………...Page 2

Appendix Figure S2. Sequence alignment and structure prediction of HDIP1/2/3……….........................Page 3

Appendix Figure S3. Phylogenetic tree of orthologues of HDIP1/2/3 in angiosperms………….................Page 4

Appendix Figure S4. Interactions of HDIP1/3, HDA19, SNL5/6, and MSI1 as detected by Y2H assays....Page 5

Appendix Figure S5. Assay for nuclear-cytoplasmic fractionation of HDIP1 and HDIP3……….................Page 6

Appendix Figure S6. Schematic diagrams of the *hdip* mutants………………………………....................Page 7

Appendix Figure S7. Morphological phenotypes of *HDIP1-Flag* complementation lines in the *hdip1/2/3* mutant background………………………………………………………………………………………………...............Page 8

Appendix Figure S8. Correlation analysis of ChIP-seq signals between HDA19 and HDIP1……..............Page 9

Appendix Figure S9. GO analysis of the HDA19 and HDIP1 co-occupied genes exhibiting increased expression in both *hda19* and *hdip1/2/3* mutants………………..Page 10

Appendix Figure S10. Determination of the role of the DNA binding domain of HDIP1 in Arabidopsis plants…………………………………………………………........……………….................................Page 11

Appendix Figure S11. Genome browser view of H3Ac ChIP-seq signals at representative ABA signaling pathway genes………………………………………......………………………………...Page 12

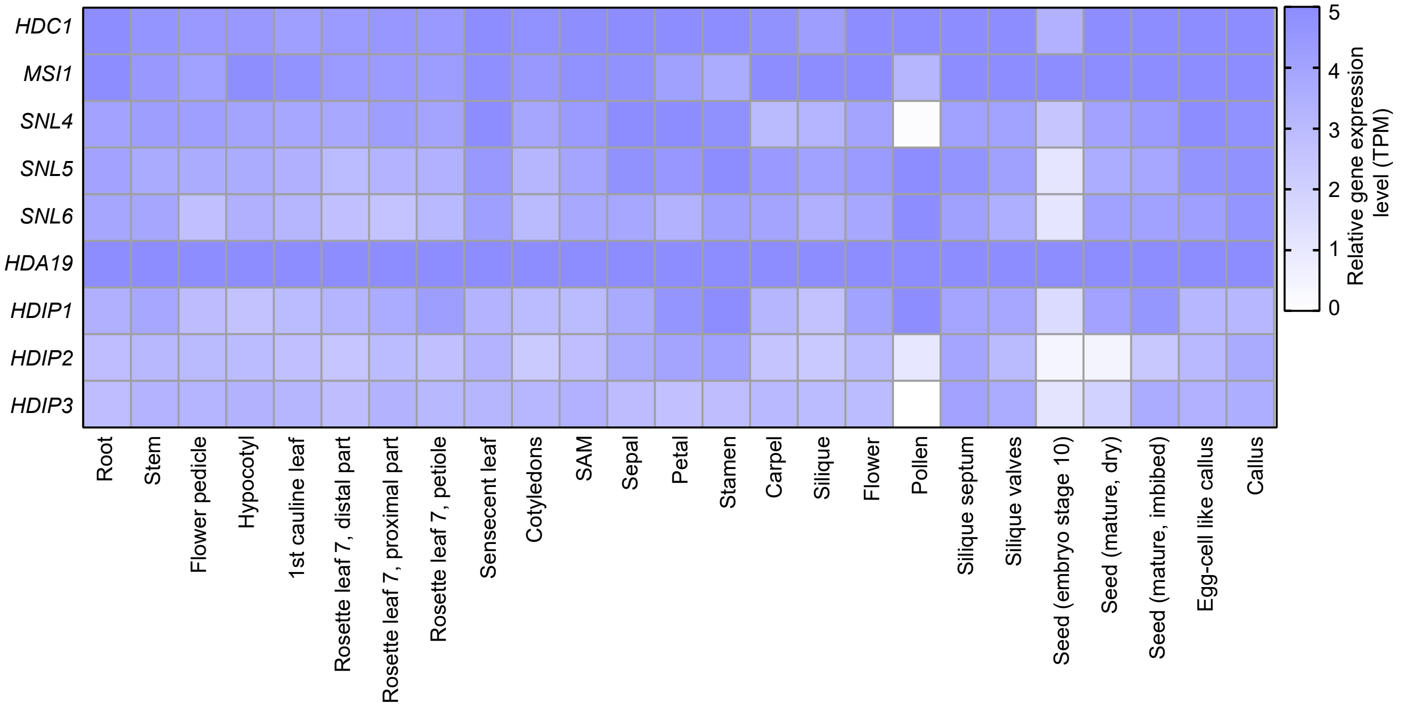

Appendix Figure S1. The expression patterns of genes encoding HDIP1/2/3 and other HDAC components in Arabidopsis.
The relative expression levels of genes encoding HDIP1/2/3 and other HDAC components were determined based on ATHENA databases (http://athena.proteomics.wzw.tum.de:5002/master_arabidopsisshiny/) in indicated tissues. TPM, transcripts per million.

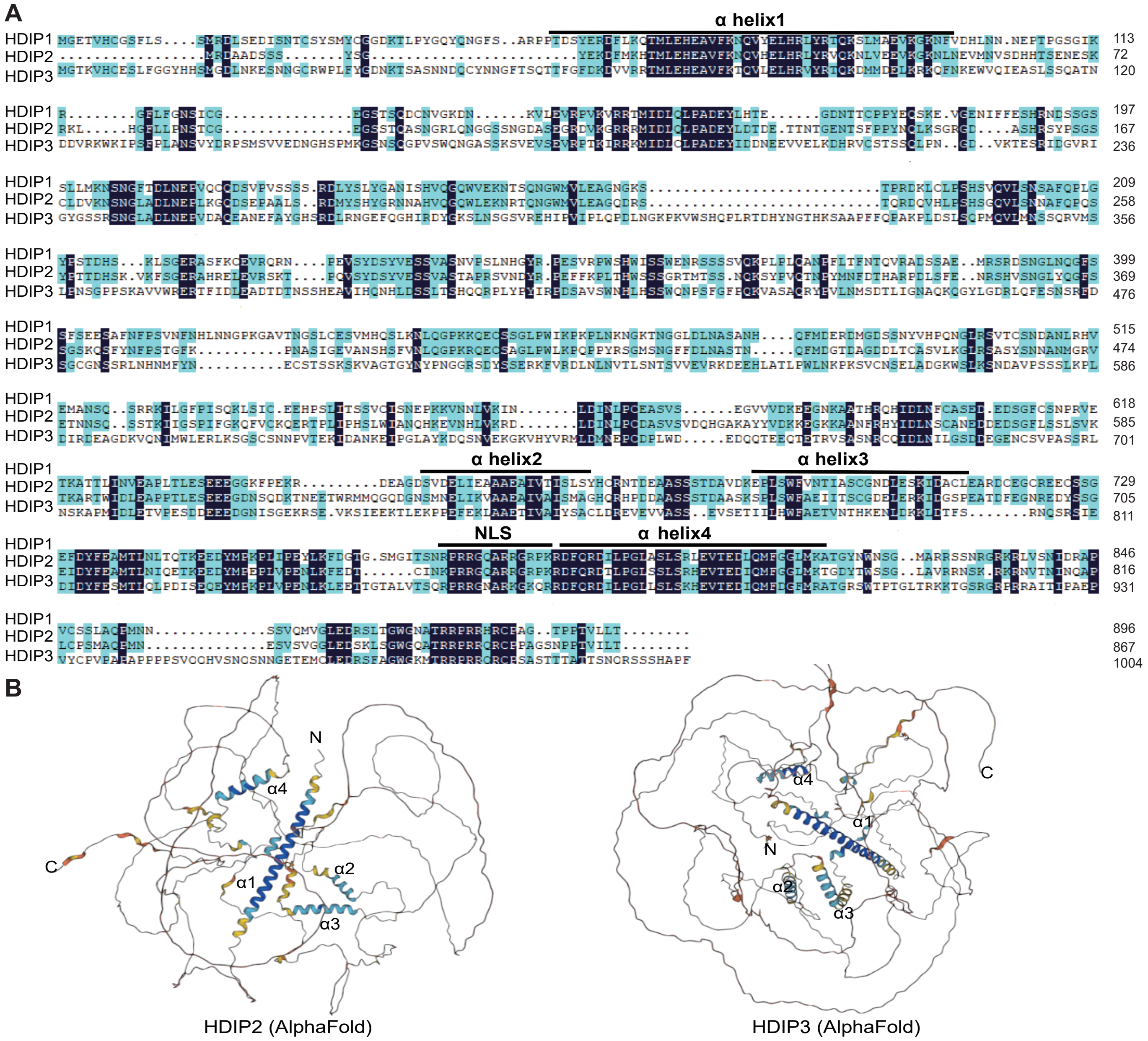

Appendix Figure S2. Sequence alignment and structure prediction of HDIP1/2/3.

(**A**) The amino acid sequence alignment of HDIP1, HDIP2, and HDIP3 proteins was performed using DNAMAN software. The black lines indicate the positions of the conserved α-helices and nuclear localization signal (NLS). The identical amino acids are highlighted in black, and the similar amino acids and partially identical amino acids are highlighted in cyan. (**B**) The structures of HDIP2 and HDIP3 were predicted by Alphafold.

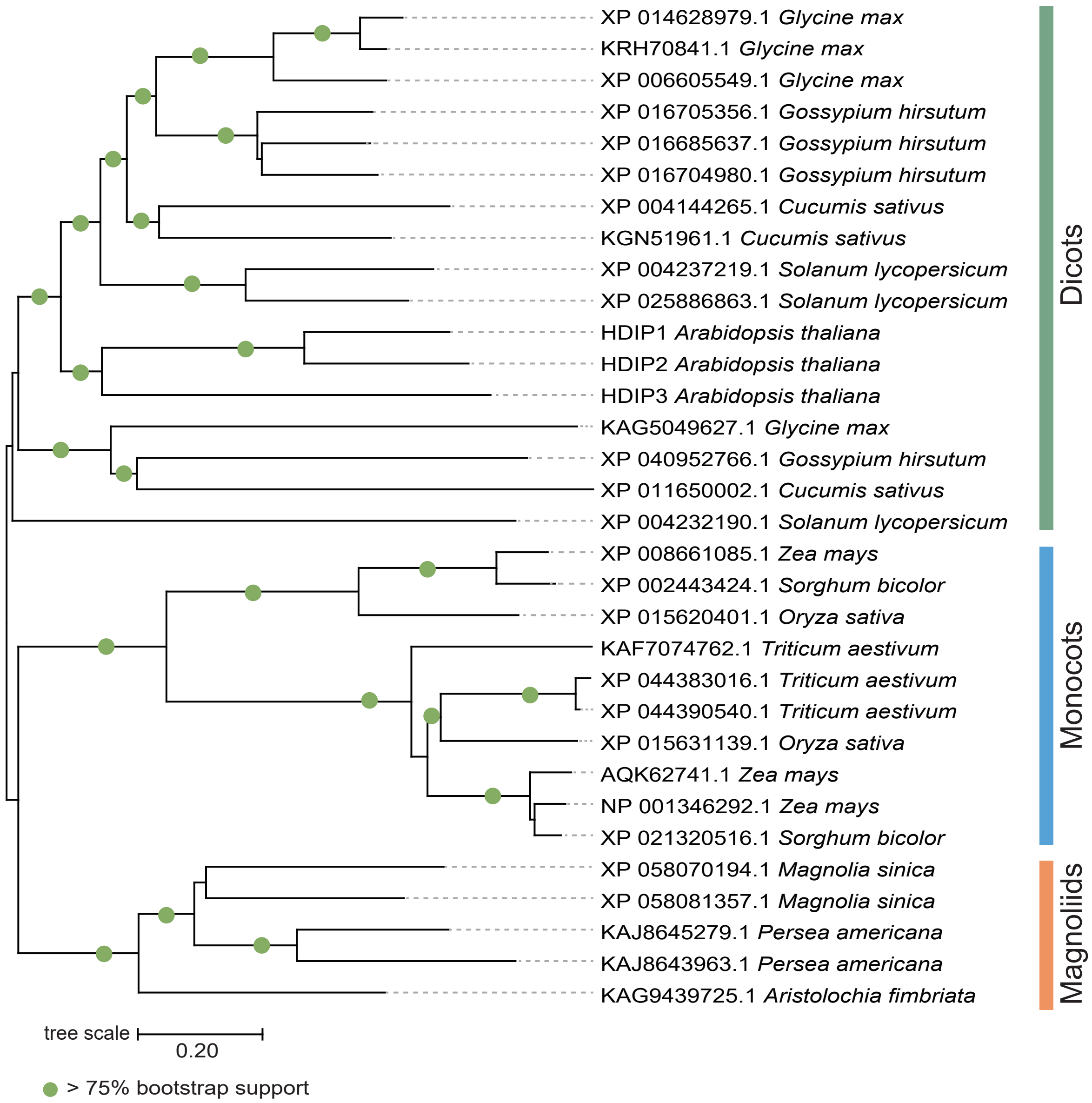

Appendix Figure S3. Phylogenetic tree of orthologues of HDIP1/2/3 in angiosperms.

The phylogenetic tree was constructed based on amino acid sequences of orthologues of HDIP1/2/3 by MEGA7 using the neighbor-joining method. The data are from monocots (blue bar), dicots (green bar) and magnoliids (orange bar). Bootstrap proportions (>75%) for each node are shown.

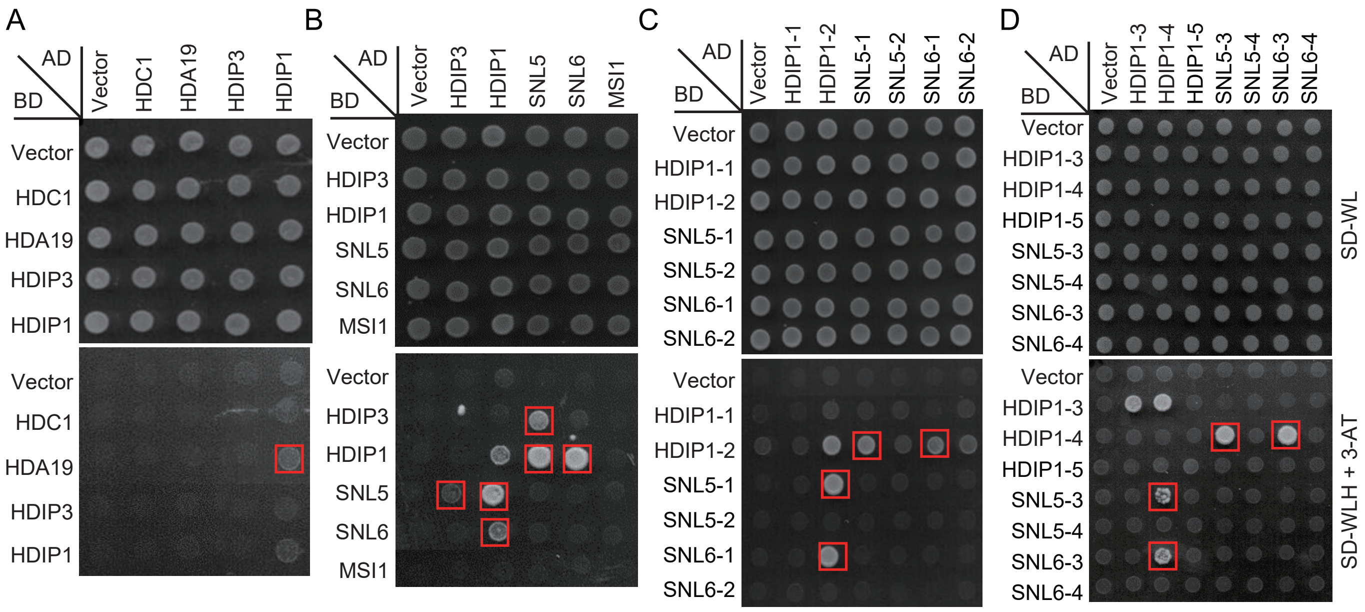

Appendix Figure S4. Interactions of HDIP1/3, HDA19, SNL5/6, and MSI1 as detected by Y2H assays.
(**A, B**) Determination of the interactions of full-length histone deacetylase complex components by Y2H assays. (**C, D**) Determination of the interactions between truncated forms of HDIP1 and truncated forms of SNL5 or SNL6 by Y2H assays. The top panels show the yeast strains grown on synthetic dropout medium lacking Trp and Leu (SD-WL), while the bottom panels indicate the yeast strains grown on synthetic dropout medium lacking Trp, Leu, and His supplemented with 3 mM 3-AT (SD-WLH + 3-AT). The interactions confirmed by pull-down assays are marked by red boxes.

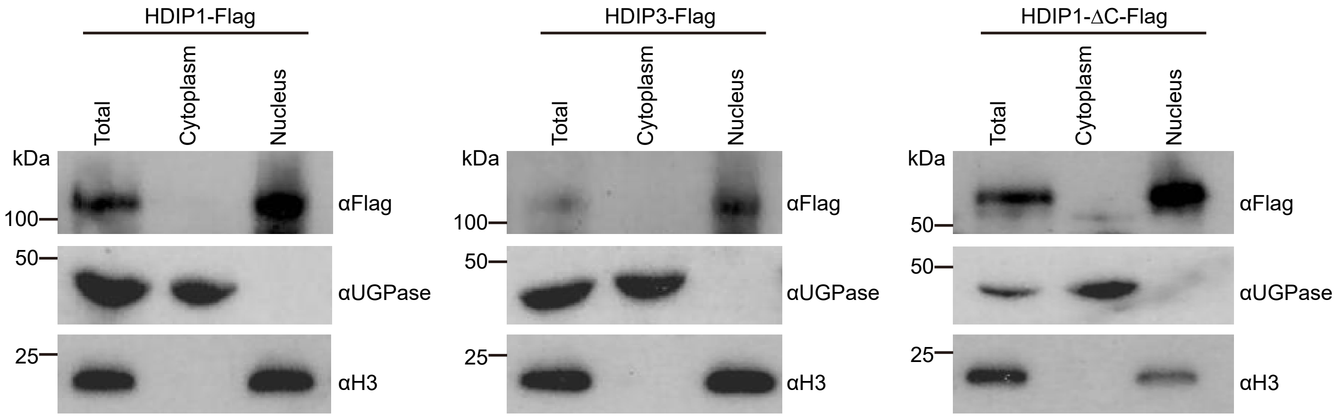

Appendix Figure S5. Assay for nuclear-cytoplasmic fractionation of HDIP1 and HDIP3.

Total proteins were isolated from transgenic plants expressing HDIP1-Flag, HDIP3-Flag, and HDIP1-ΔC-Flag, followed by nuclear-cytoplasmic fractionation. The HDIP1-Flag, HDIP3-Flag, and HDIP1-ΔC-Flag proteins were detected in the total protein, nucleus, and cytoplasmic fractions by western blot analysis with Flag antibody. The histone H3 and UGPase signals were shown as indicators for nuclear and cytoplasmic proteins, respectively.

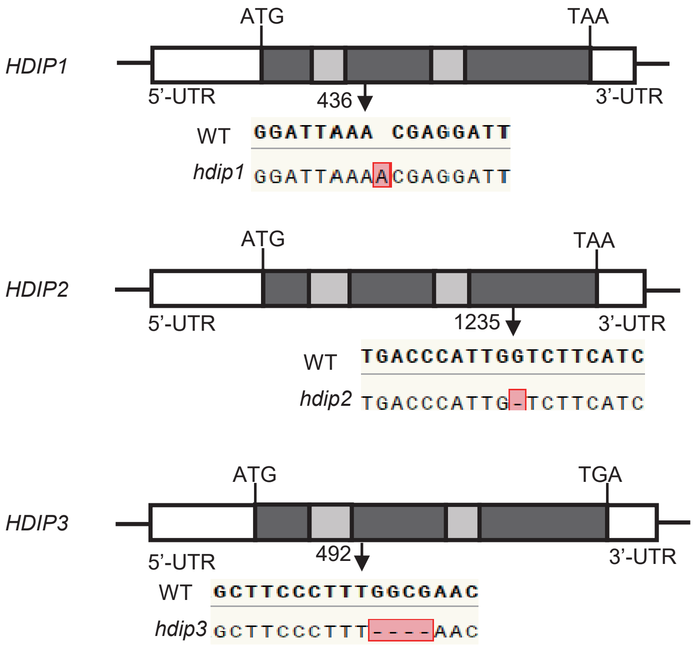

Appendix Figure S6. Schematic diagrams of the *hdip* mutants.

The mutations in the various *hdip* mutants were generated through the CRISPR-Cas9 genome editing system. Within the gene diagrams, the dark gray, light gray, and white boxes represent exons, introns, and the 5'-UTR or 3'-UTR, respectively. The sequences of the *HDIP1*, *HDIP2*, and *HDIP3* genes in the mutants are aligned with those in the wild-type plants, with the mutations being marked with red boxes.

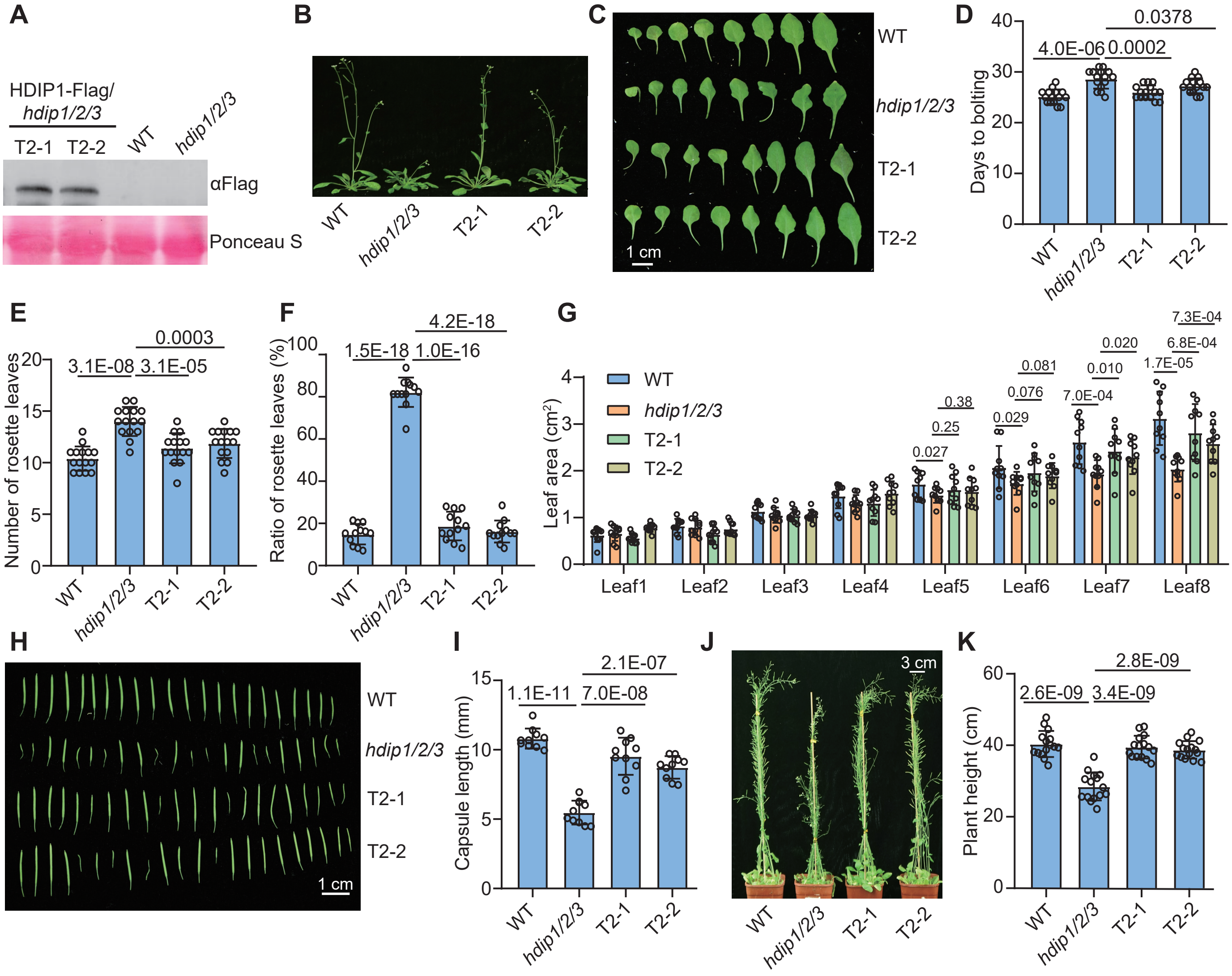

Appendix Figure S7. Morphological phenotypes of *HDIP1-Flag* complementation lines in the *hdip1/2/3* mutant background.
(**A**) Determination of the expression of the *HDIP1-Flag* in the *hdip1/2/3* mutant background by immunoblotting. The data are derived from two complementation lines, T2-1 and T2-2. Ponceau S-stained ribosome proteins are shown as a loading control. (**B, C**) Morphological phenotypes of flowering time (B), and detached rosette leaves from adult plants (C). (**D-G**) Statistical analyses of the days to bolting (n = 15) (D), the number of rosette leaves (n = 15) (E), ratio of curved rosette leaves (n = 12) (F), and the leaf area (n = 10) (G). Data are mean ± SD. *P* values were determined by two-tailed Student's *t*-test. (**H, I**) Morphological phenotype (H) and length analysis (I) of siliques in the wild type, *hdip1/2/3*, and two independent complementation lines. Data are mean ± SD (n = 10). *P* values were determined by two-tailed Student's *t*-test. (**J, K**) Plant height phenotype (J) and statistical analysis (K) of the wild type, *hdip1/2/3*, and two independent complementation lines. Data are mean ± SD (n = 15). *P* values were determined by two-tailed Student's *t*-test.

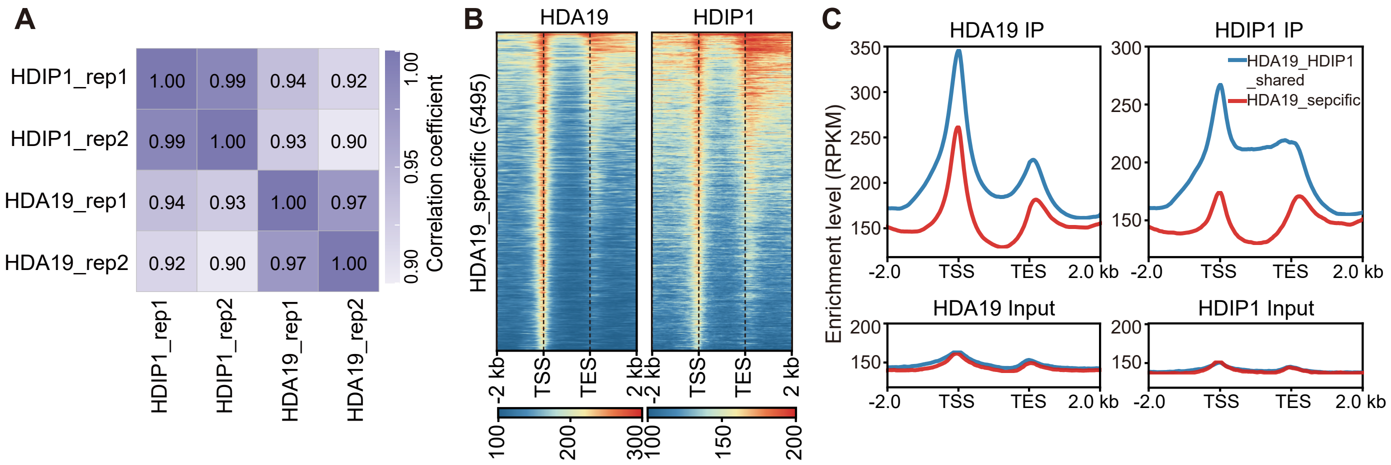

Appendix Figure S8. Correlation analysis of ChIP-seq signals between HDA19 and HDIP1.

(**A**) Metaplots showing correlation of ChIP-seq signals between HDA19 and HDIP1. Data are from two biological replicates. (**B**) Heatmaps showing the ChIP-seq signals of HDA19-specific target genes (5,495). The HDA19-specific target genes were sorted by the HDA19 enrichment level. The scale represents RPKM. (**C**) Meta plots showing the ChIP-seq signals of HDA19 and HDIP1 over HDA19- and HDIP1-shared target genes and HDA19-specific genes. TSS, transcription start site; TES, transcription end site. "-2 kb" and "2 kb" represent the 2-kb regions upstream of TSS and downstream of TES, respectively.

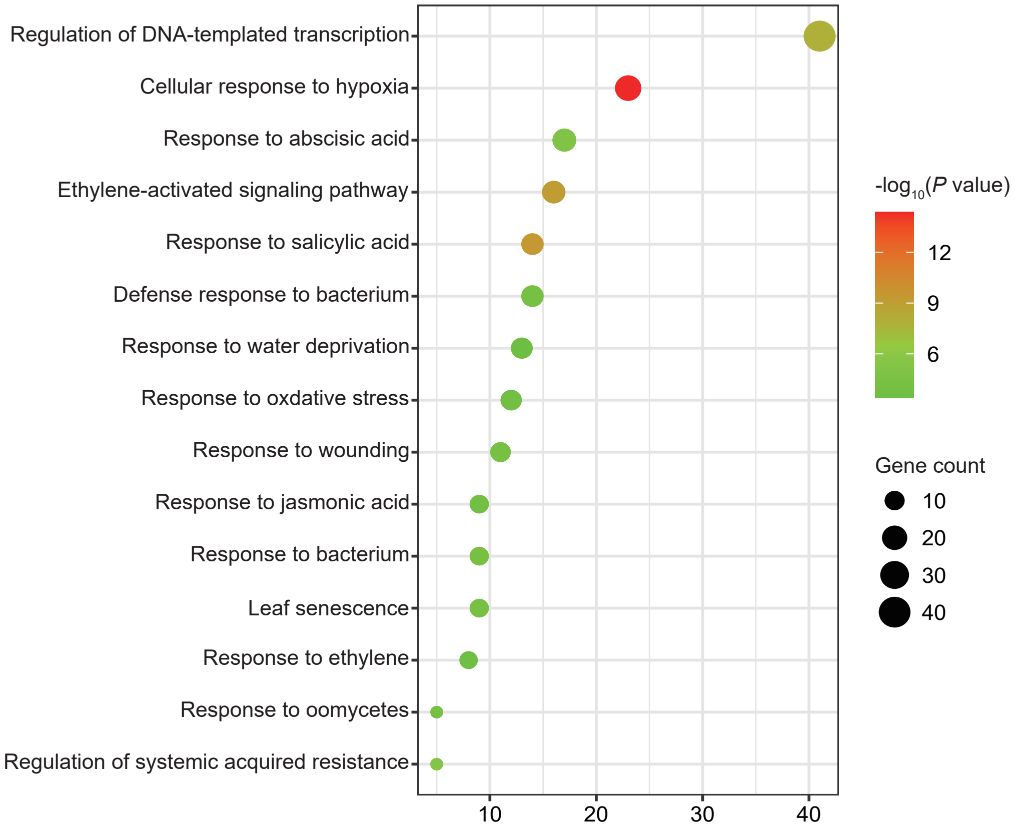

Appendix Figure S9. GO analysis of the HDA19 and HDIP1 co-occupied genes exhibiting increased expression in both *hda19* and *hdip1/2/3* mutants.

The overlap between the HDA19 and HDIP1 co-occupied genes and the genes exhibiting increased expression in both *hda19* and *hdip1/2/3* mutants were subjected to the GO analysis. The GO analysis was performed by the online DAVID 2023 tool. *P* values were determined by one-sided Fisher's Exact test.

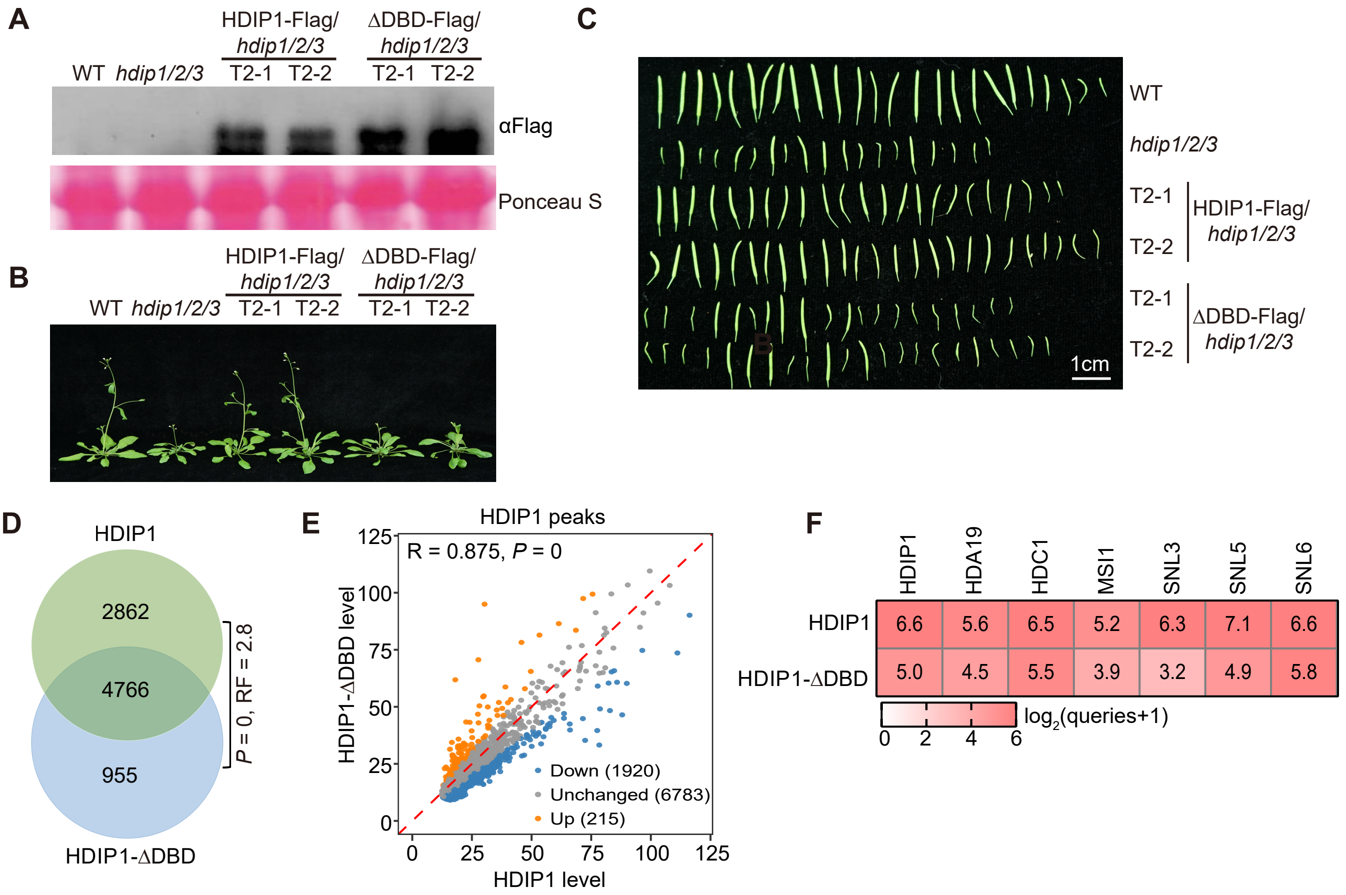

Appendix Figure S10. Determination of the role of the DNA binding domain of HDIP1 in Arabidopsis plants.
(**A**) The expression of Flag-tagged wild-type HDIP1 and HDIP1-ΔDBD proteins in the *hdip1/2/3* mutant background detected by western blot analysis. The data are derived from two complementation lines, T2-1 and T2-2. Ponceau S-stained ribosome proteins are shown as a loading control. (**B, C**) Morphological phenotype of wild type, *hdip1/2/3*, and the complementation lines expressing wild-type HDIP1 and HDIP1-ΔDBD. The phenotype of flowering time (B) and siliques (C) are shown. (**D**) Venn diagram showing the overlap between HDIP1 target genes and HDIP1-ΔDBD target genes. *P* values determined by the hypergeometric test (one-tailed). (**E**) Scatter plot showing the correlation of HDIP1-Flag and HDIP1-ΔDBD ChIP-seq signals at HDIP1 target genes. The Pearson correlation coefficient (R) and the significance of the correlation (*P* values) are shown. (**F**) Identification of proteins that interact with HDIP1-Flag and HDIP1-ΔDBD-Flag in Arabidopsis plants by AP-MS. The number represents the normalization of matched queries.

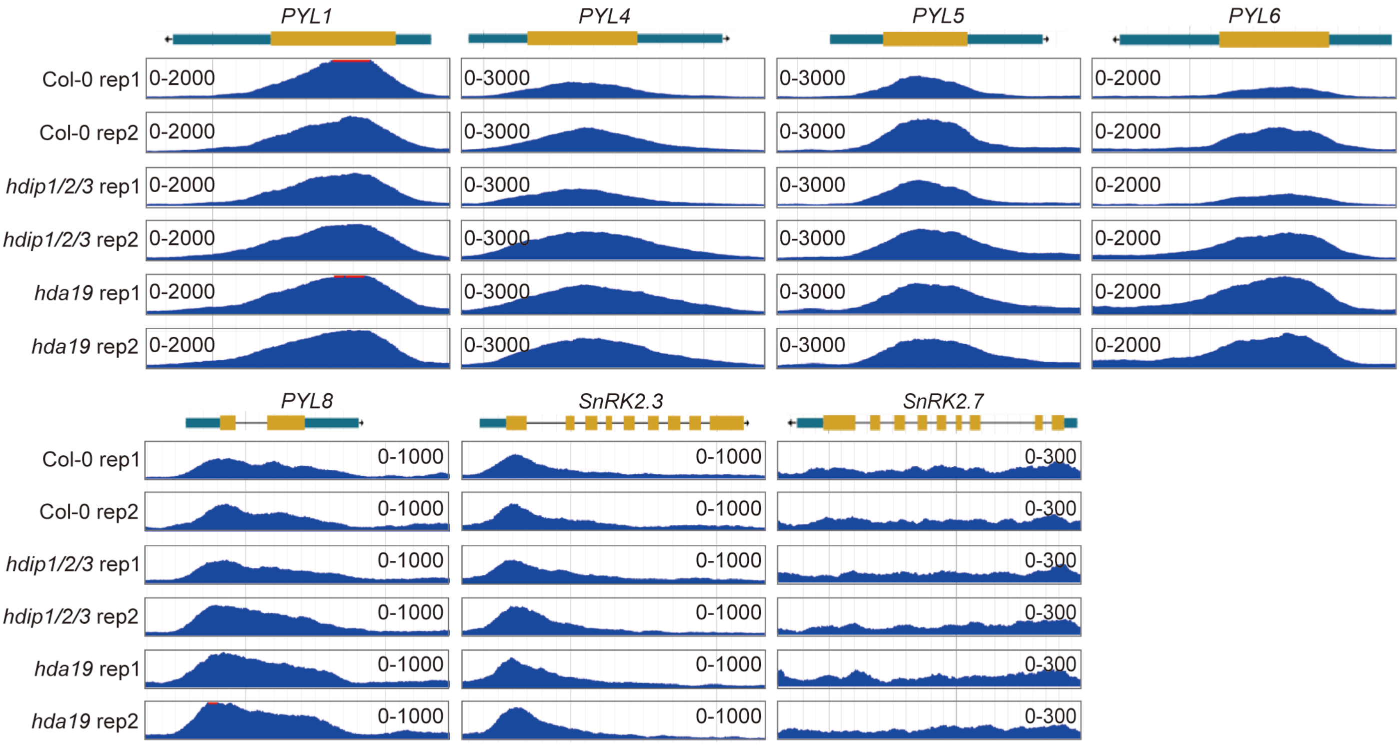

Appendix Figure S11. Genome browser view of H3Ac ChIP-seq signals at representative ABA signaling pathway genes.
The H3Ac ChIP-seq signals are shown in the wild-type Col-0, *hda19*, and *hdip1/2/3* mutants. Two independent replicates of the ChIP-seq results are shown. The scale represents RPKM.
