# Supplementary figures and images for: Essential angiosperm-specific subunits of HDA19 histone deacetylase complexes in Arabidopsis
Source: EMBO J. 2025 Apr 28;44(12):3521–46. doi: 10.1038/s44318-025-00445-w (PMC12170880; doi:10.1038/s44318-025-00445-w)

Fig 1D

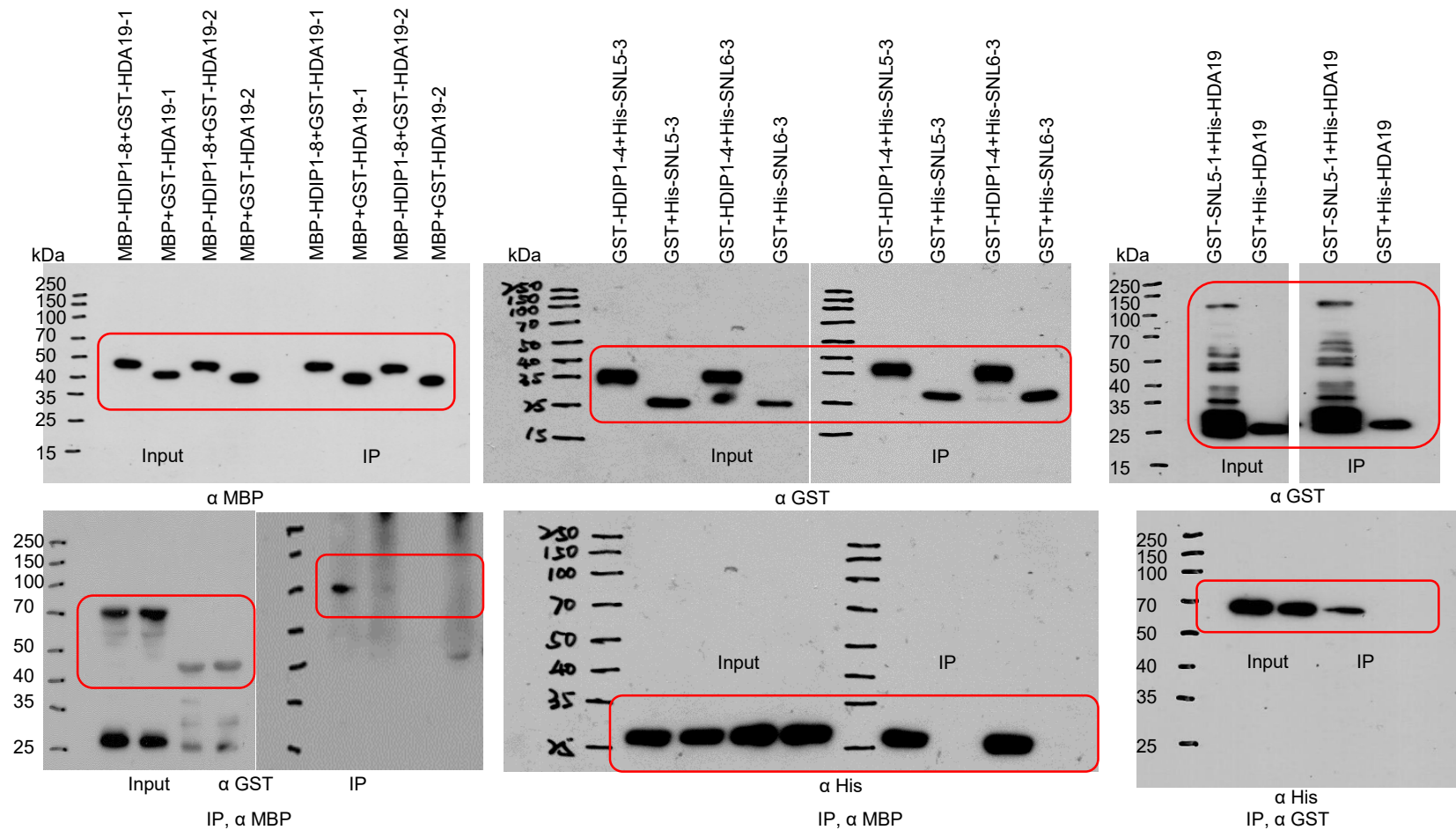

Supplement: Supplementary file 8 — Source data Fig. 1 [file 44318_2025_445_MOESM8_ESM.zip › Fig 1D Source Data.pdf]

Fig 2A-C

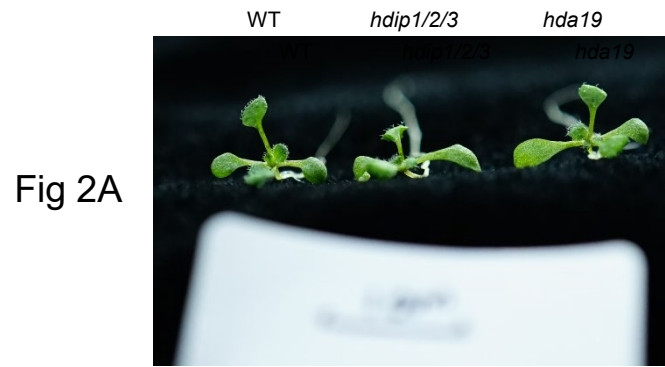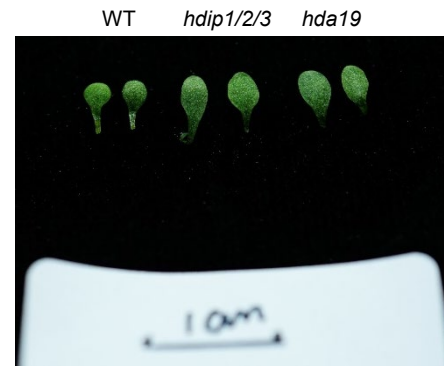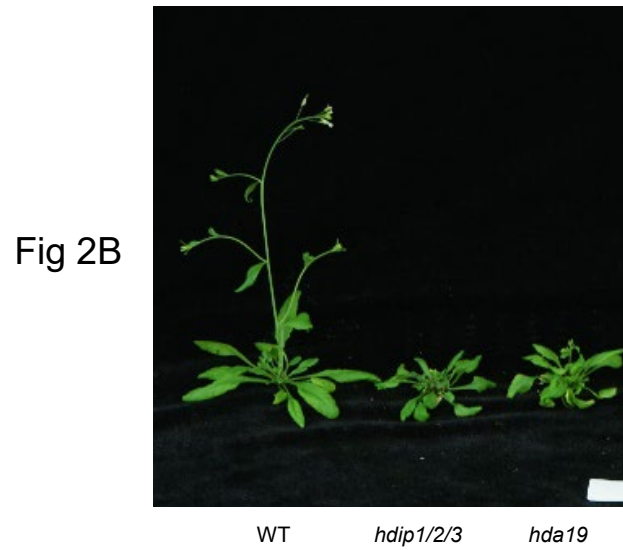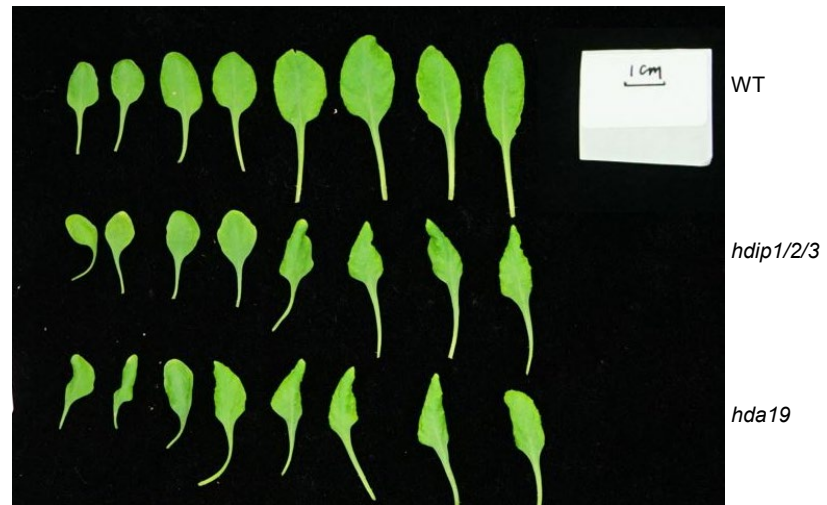

Supplement: Supplementary file 9 — Source data Fig. 2 [file 44318_2025_445_MOESM9_ESM.zip › Fig 2A-C Source Data.pdf]

Fig 2D-G

Fig 2D

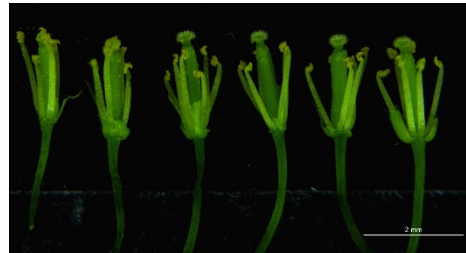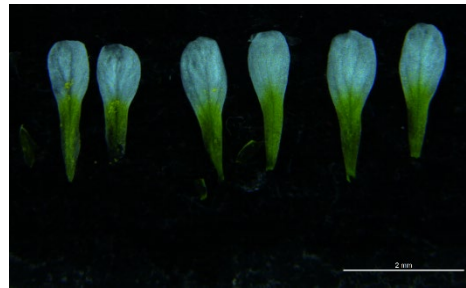

WT

*hdip1/2/3*

*hda19*

Fig 2E

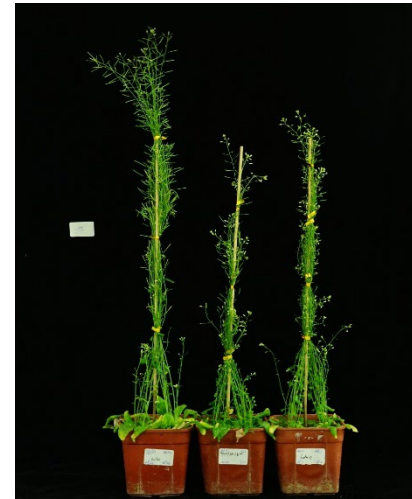

WT

*hdip1/2/3*

*hda19*

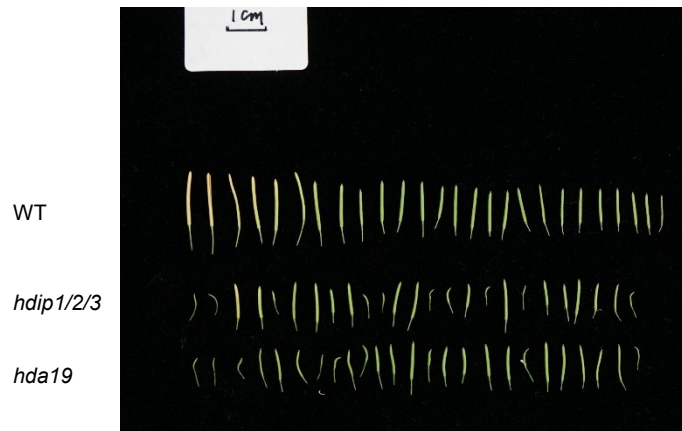

WT

*hdip1/2/3*

*hda19*

Fig 2F

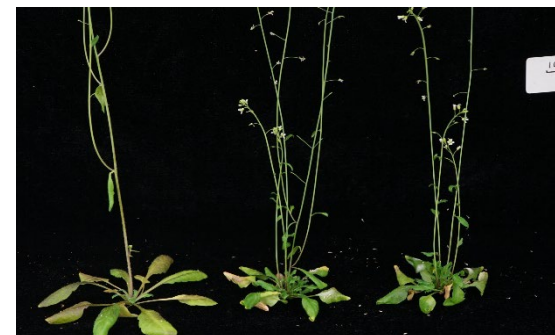

WT

*hdip1/2/3*

*hda19*

Fig 2G

Supplement: Supplementary file 9 — Source data Fig. 2 [file 44318_2025_445_MOESM9_ESM.zip › Fig 2D-G Source Data.pdf]

Fig 6B, 6C

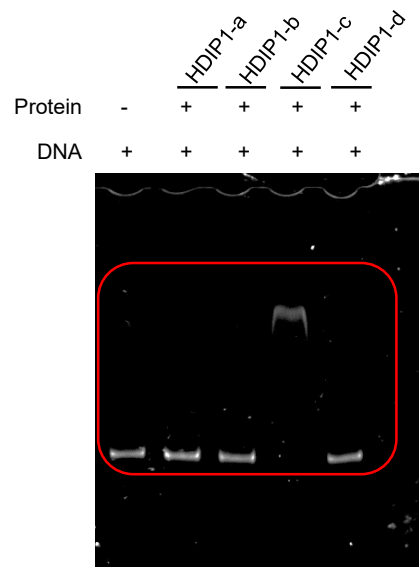

Fig 6B

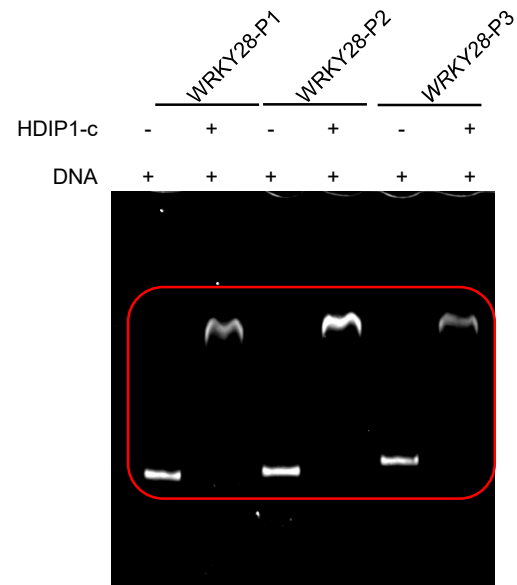

Fig 6C

Supplement: Supplementary file 11 — Source data Fig. 6 [file 44318_2025_445_MOESM11_ESM.zip › Fig 6B 6C Source Data.pdf]

Fig 6D, 6E

|          |                         |   |   |   |   |                         |   |   |   |   |
|----------|-------------------------|---|---|---|---|-------------------------|---|---|---|---|
|          | 1.2 2.4 3.6 4.8 $\mu$ g |   |   |   |   | 1.2 2.4 3.6 4.8 $\mu$ g |   |   |   |   |
| Protein  | -                       | + | + | + | + | -                       | + | + | + | + |
| P1-1 DNA | +                       | + | + | + | + | -                       | - | - | - | - |
| P1 DNA   | -                       | - | - | - | - | +                       | + | + | + | + |

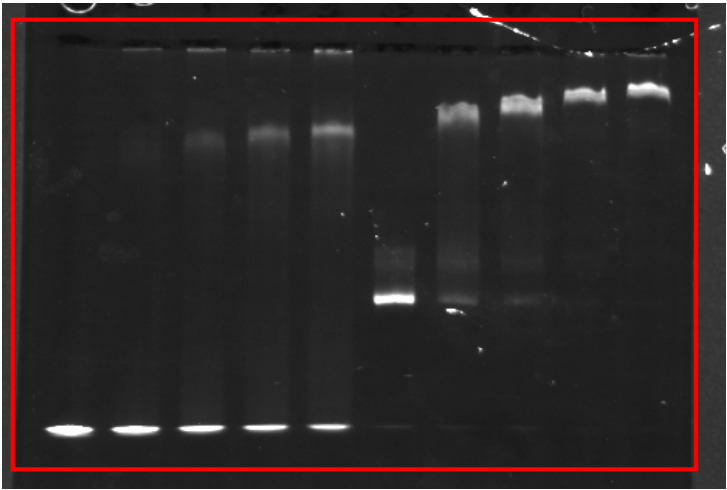

Fig 6D

|          |   |   |     |     |   |   |     |    |     |       |
|----------|---|---|-----|-----|---|---|-----|----|-----|-------|
| P1 DNA   | 0 | 0 | 2.5 | 2.5 | 5 | 5 | 7.5 | 10 | 7.5 | 10 ng |
| P1-1 DNA | + | + | +   | +   | + | + | +   | +  | +   | +     |
| HDIP1-c  | - | + | -   | +   | - | + | -   | -  | +   | +     |

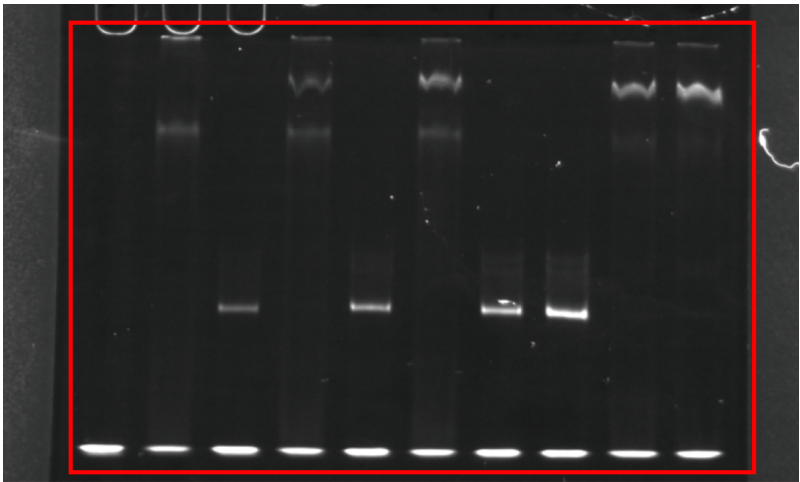

Fig 6E

Supplement: Supplementary file 11 — Source data Fig. 6 [file 44318_2025_445_MOESM11_ESM.zip › Fig 6D 6E Source Data.pdf]

Fig 6G, 6H

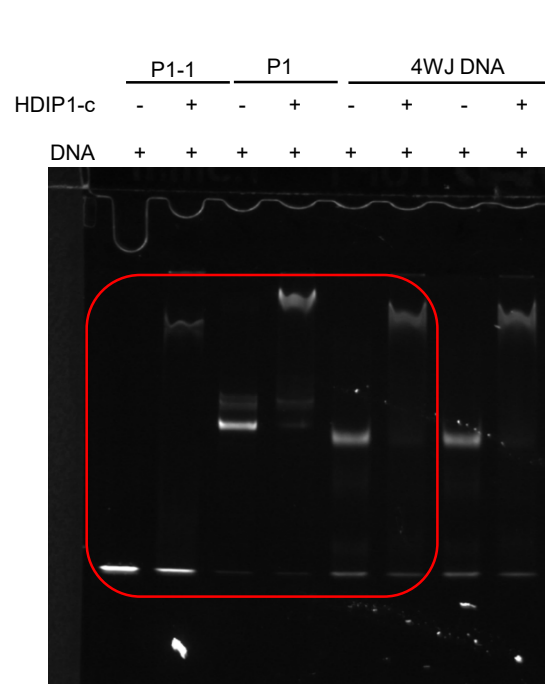

Fig 6G

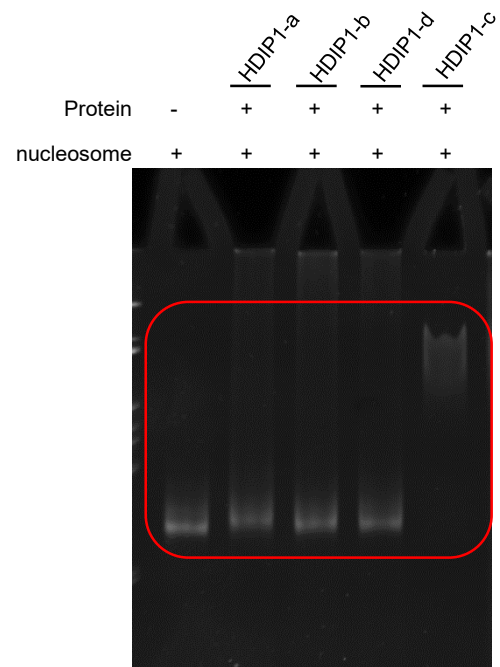

Fig 6H

Supplement: Supplementary file 11 — Source data Fig. 6 [file 44318_2025_445_MOESM11_ESM.zip › Fig 6G 6H Source Data.pdf]

Fig 7A

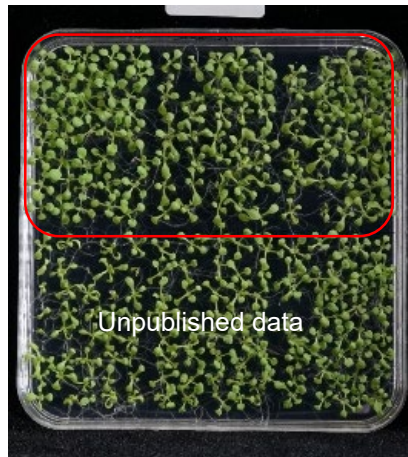

1/2 MS

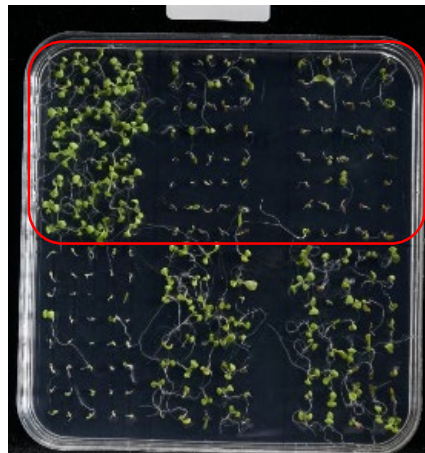

0.2  $\mu$ M ABA

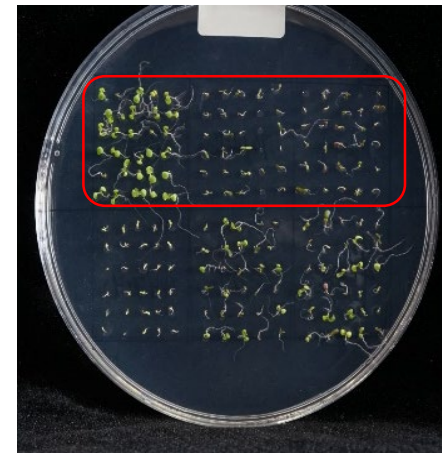

0.4  $\mu$ M ABA

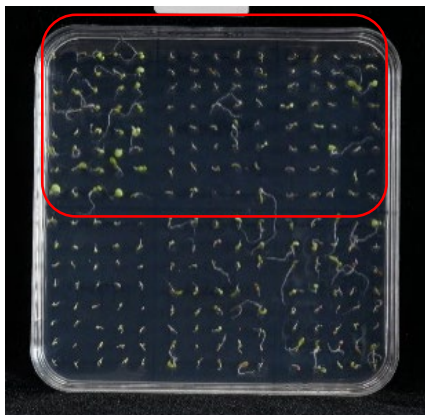

0.6  $\mu$ M ABA

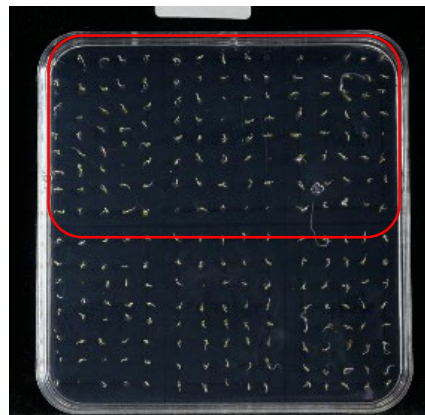

0.8  $\mu$ M ABA

|    |                  |              |
|----|------------------|--------------|
| WT | <i>hdip1/2/3</i> | <i>hda19</i> |
|----|------------------|--------------|

Supplement: Supplementary file 12 — Source data Fig. 7 [file 44318_2025_445_MOESM12_ESM.zip › Fig 7A Source Data.pdf]

Fig 7C

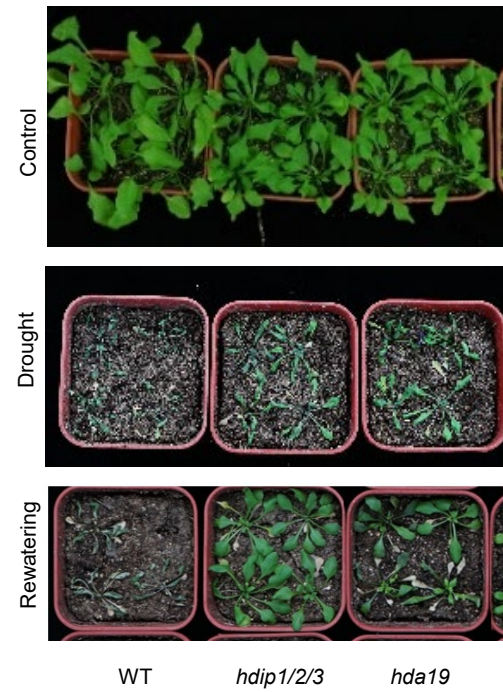

Supplement: Supplementary file 12 — Source data Fig. 7 [file 44318_2025_445_MOESM12_ESM.zip › Fig 7C Source Data.pdf]

Fig EV1E

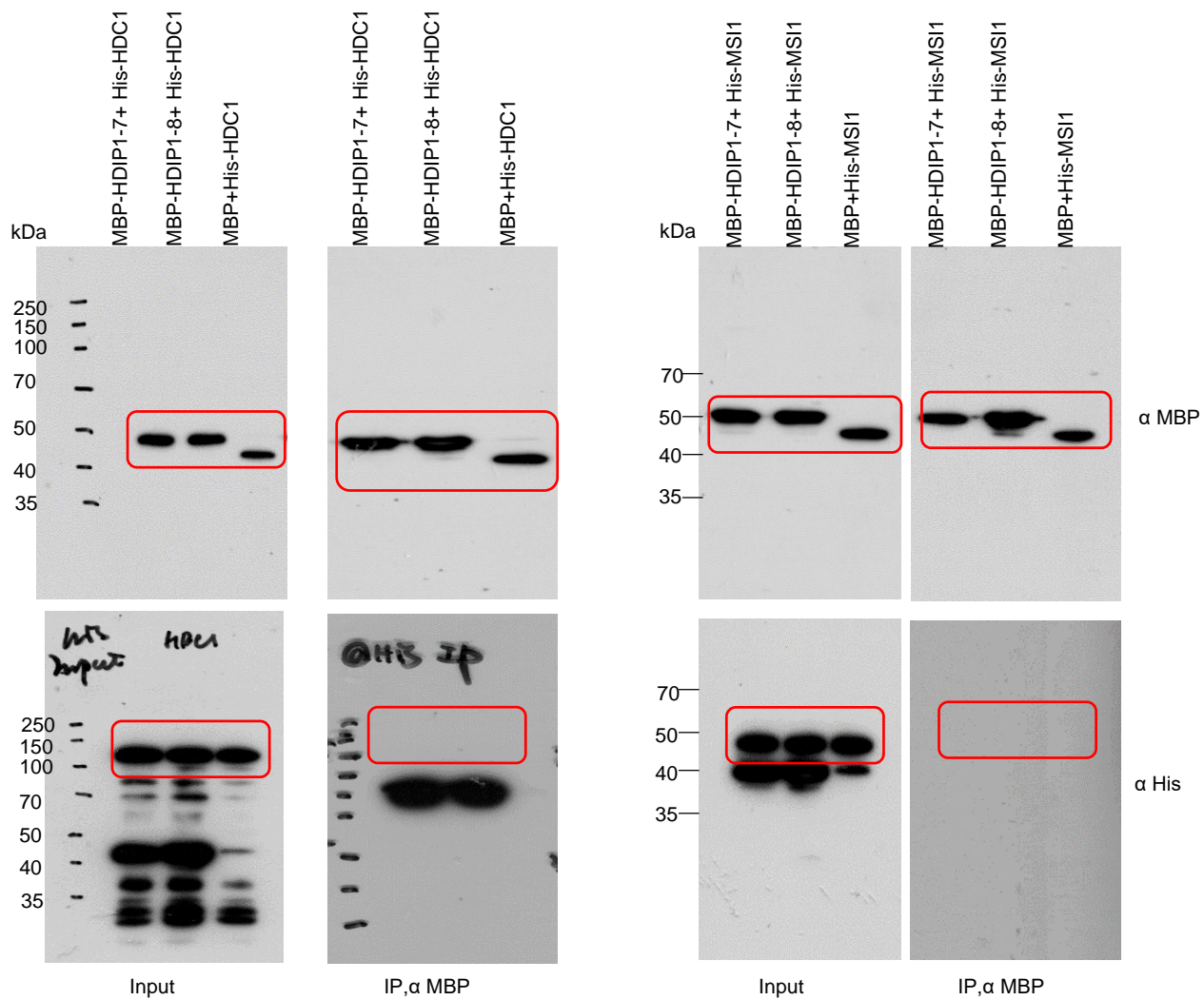

Supplement: Supplementary file 13 — Figure EV1 Source Data [file 44318_2025_445_MOESM13_ESM.zip › Fig EV1E Source Data.pdf]

Fig EV1A

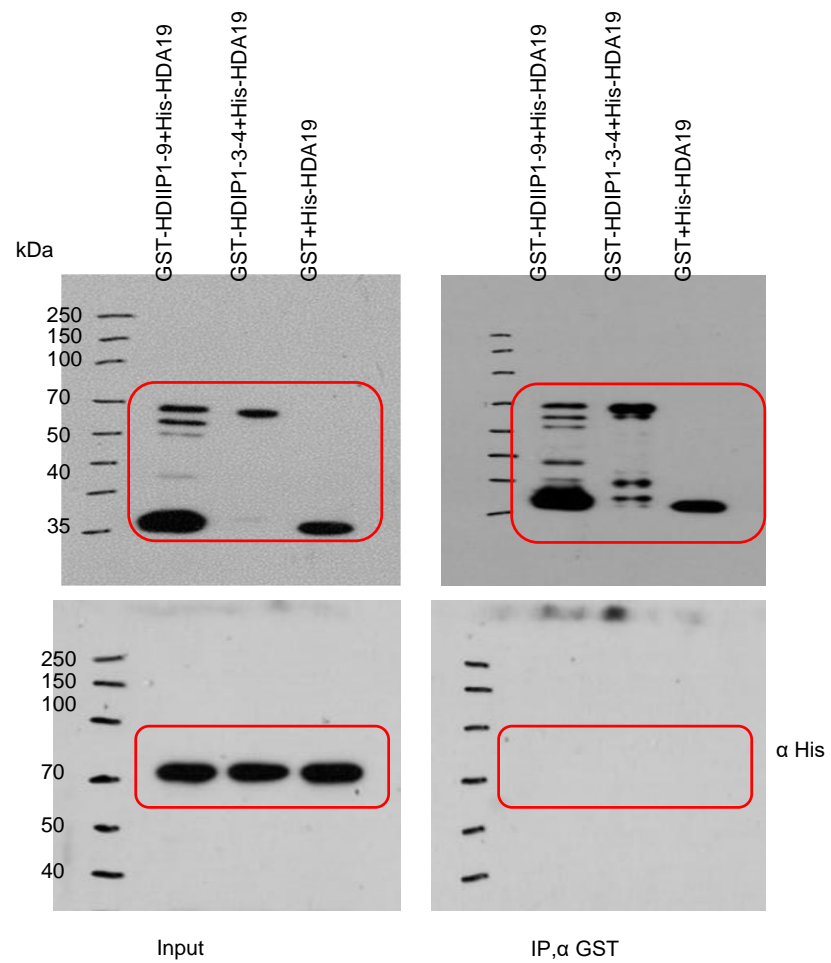

Supplement: Supplementary file 13 — Figure EV1 Source Data [file 44318_2025_445_MOESM13_ESM.zip › Fig EV1A Source Data.pdf]

Fig EV1B

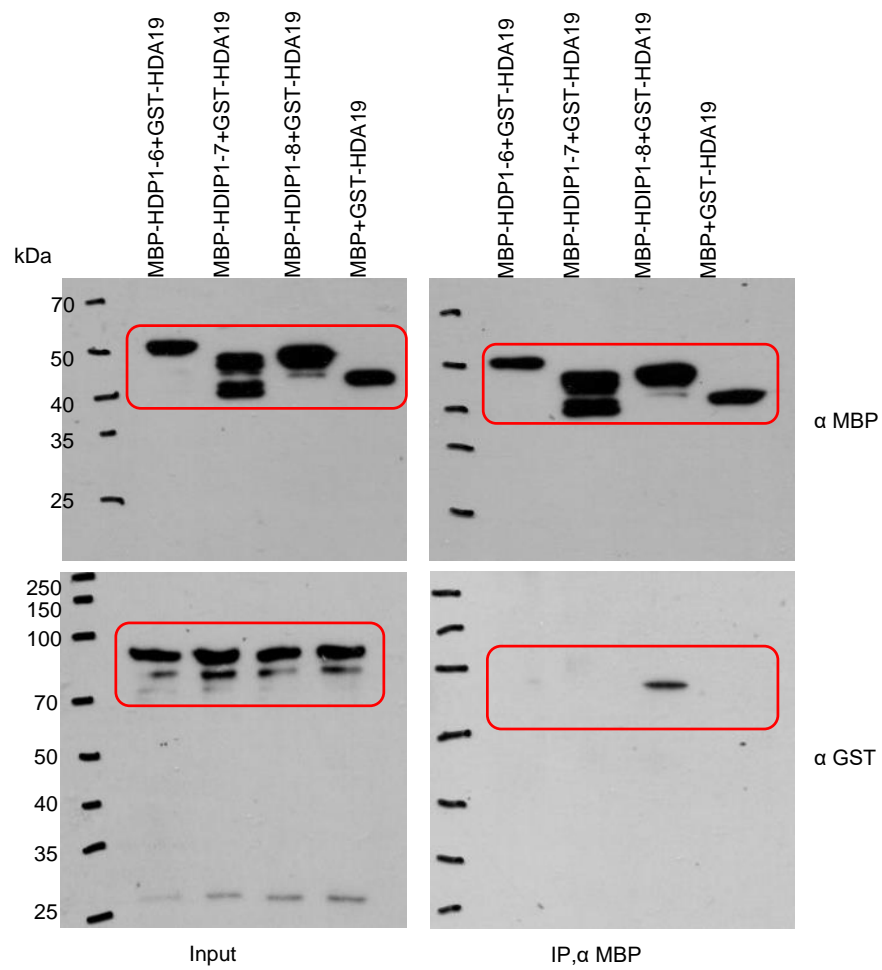

Supplement: Supplementary file 13 — Figure EV1 Source Data [file 44318_2025_445_MOESM13_ESM.zip › Fig EV1B Source Data.pdf]

Fig EV1C

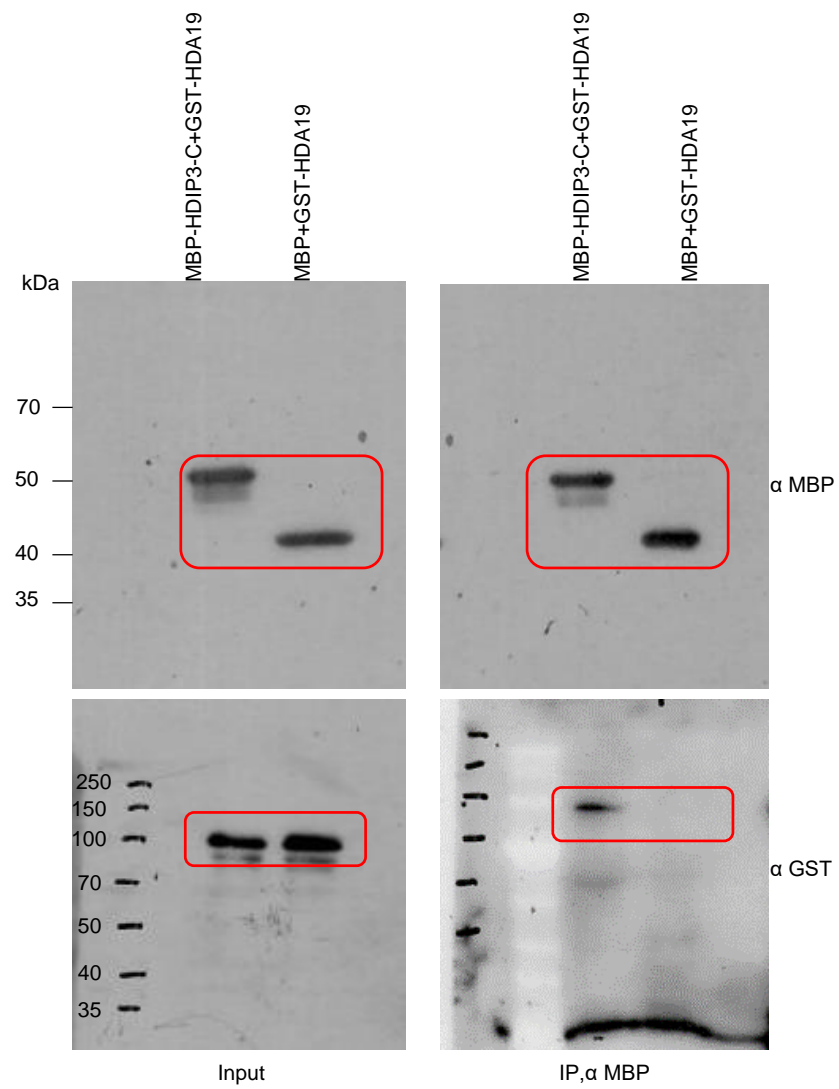

Supplement: Supplementary file 13 — Figure EV1 Source Data [file 44318_2025_445_MOESM13_ESM.zip › Fig EV1C Source Data.pdf]

Fig EV1D

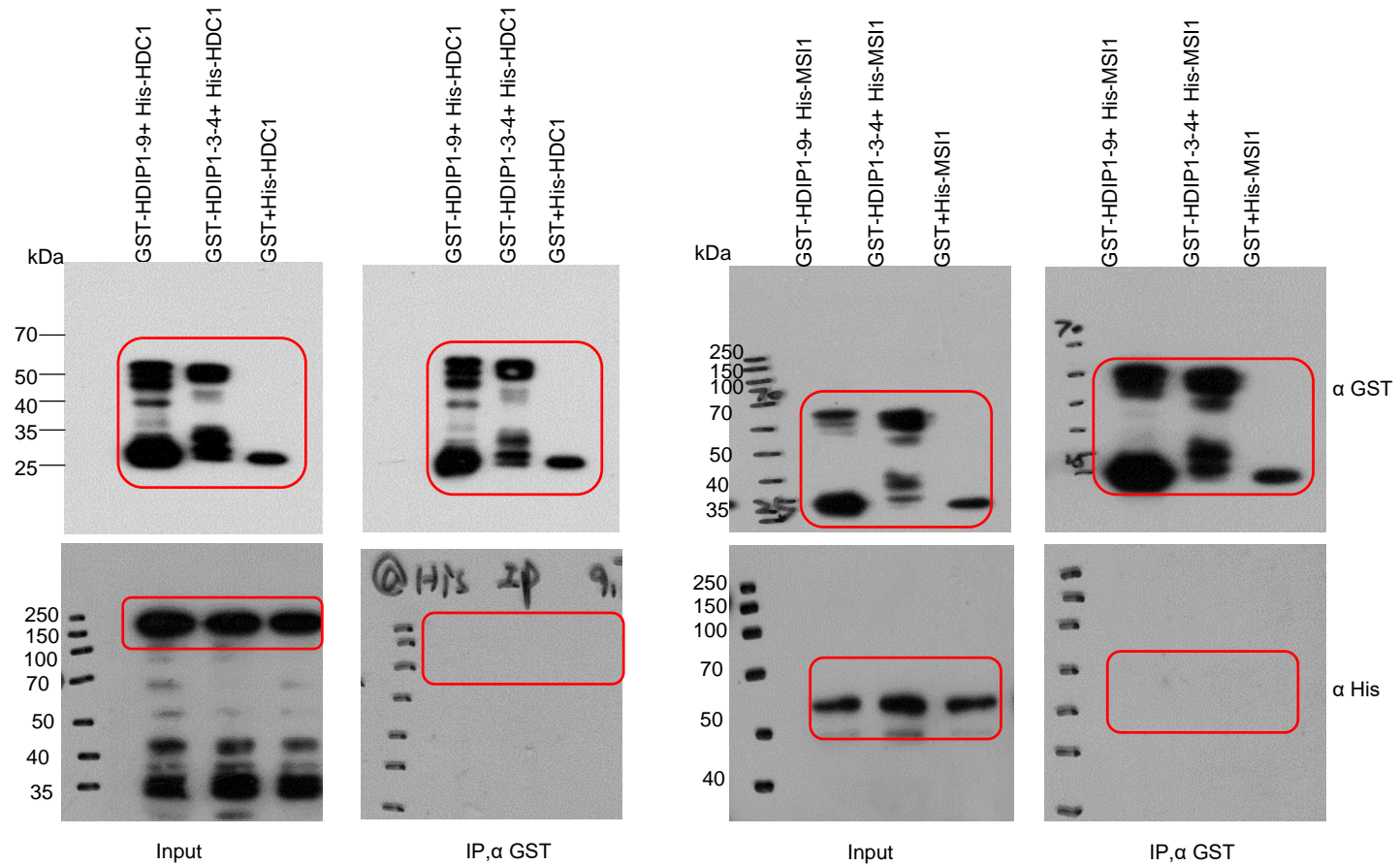

Supplement: Supplementary file 13 — Figure EV1 Source Data [file 44318_2025_445_MOESM13_ESM.zip › Fig EV1D Source Data.pdf]

Fig EV2A

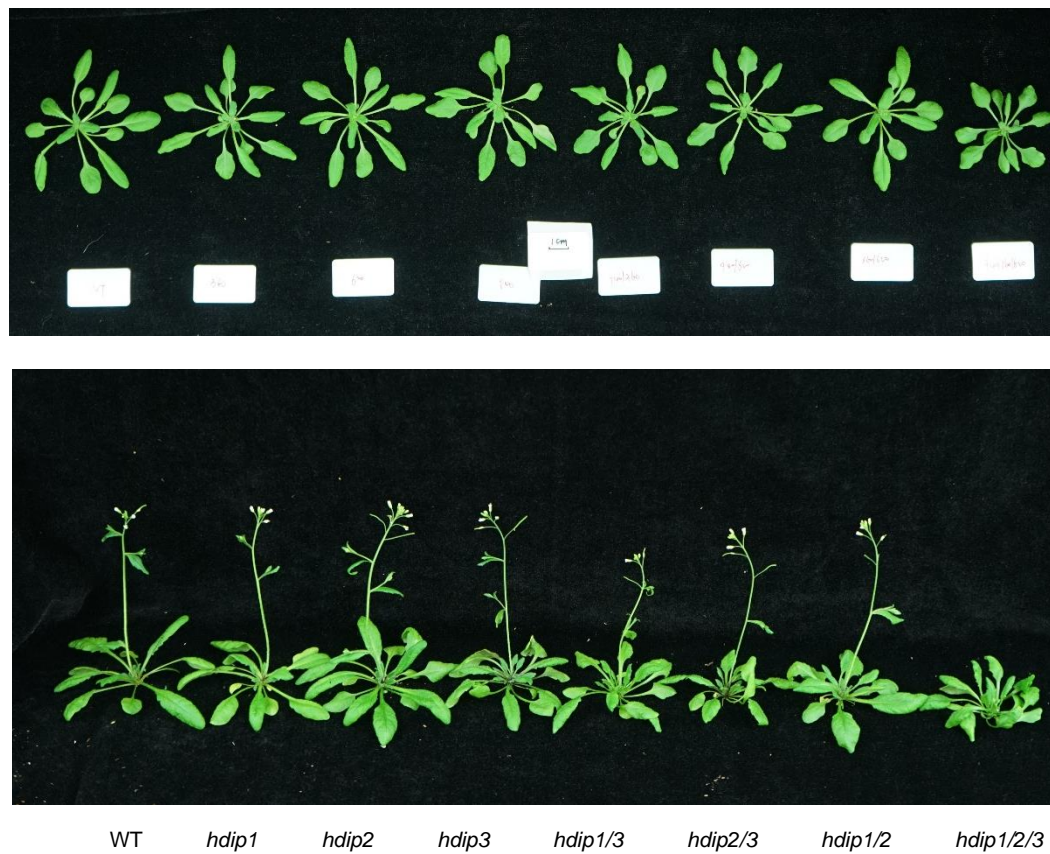

Supplement: Supplementary file 14 — Figure EV2 Source Data [file 44318_2025_445_MOESM14_ESM.zip › Fig EV2A Source Data.pdf]

Fig EV2D, 2G, 2I

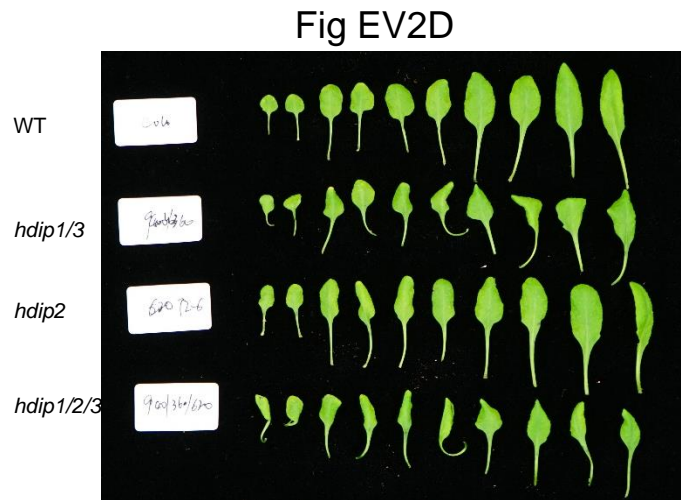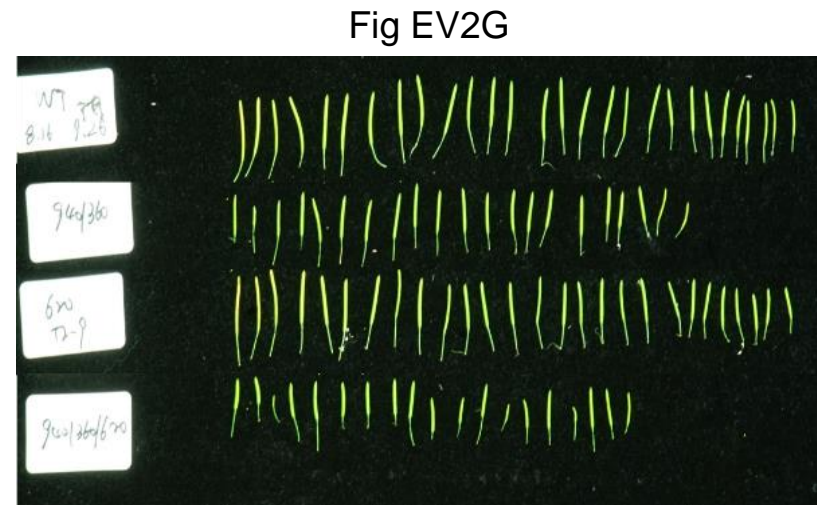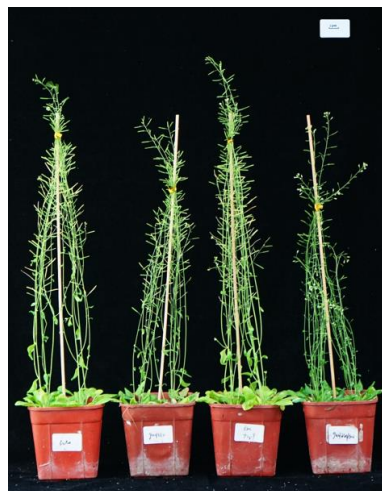

WT *hdip1/3* *hdip2* *hdip1/2/3*

Fig EV2I

Supplement: Supplementary file 14 — Figure EV2 Source Data [file 44318_2025_445_MOESM14_ESM.zip › Fig EV2D 2G 2I Source Data.pdf]

Fig EV3E

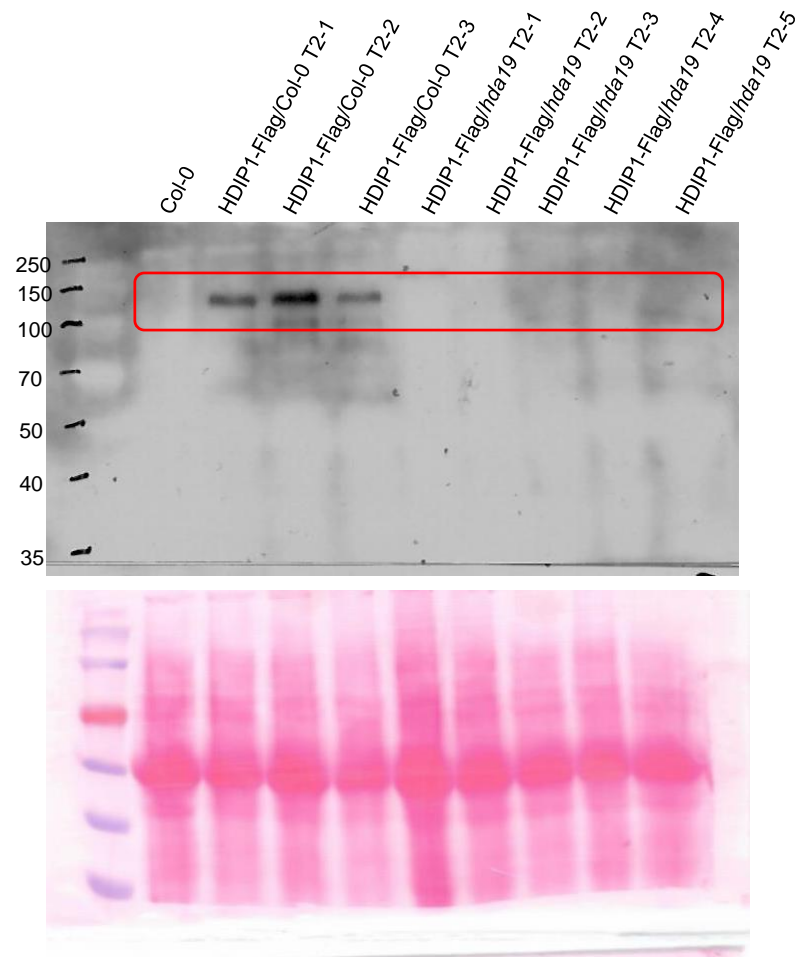

Supplement: Supplementary file 15 — Figure EV3 Source Data [file 44318_2025_445_MOESM15_ESM.zip › Fig EV3E Source Data.pdf]

Fig EV4A

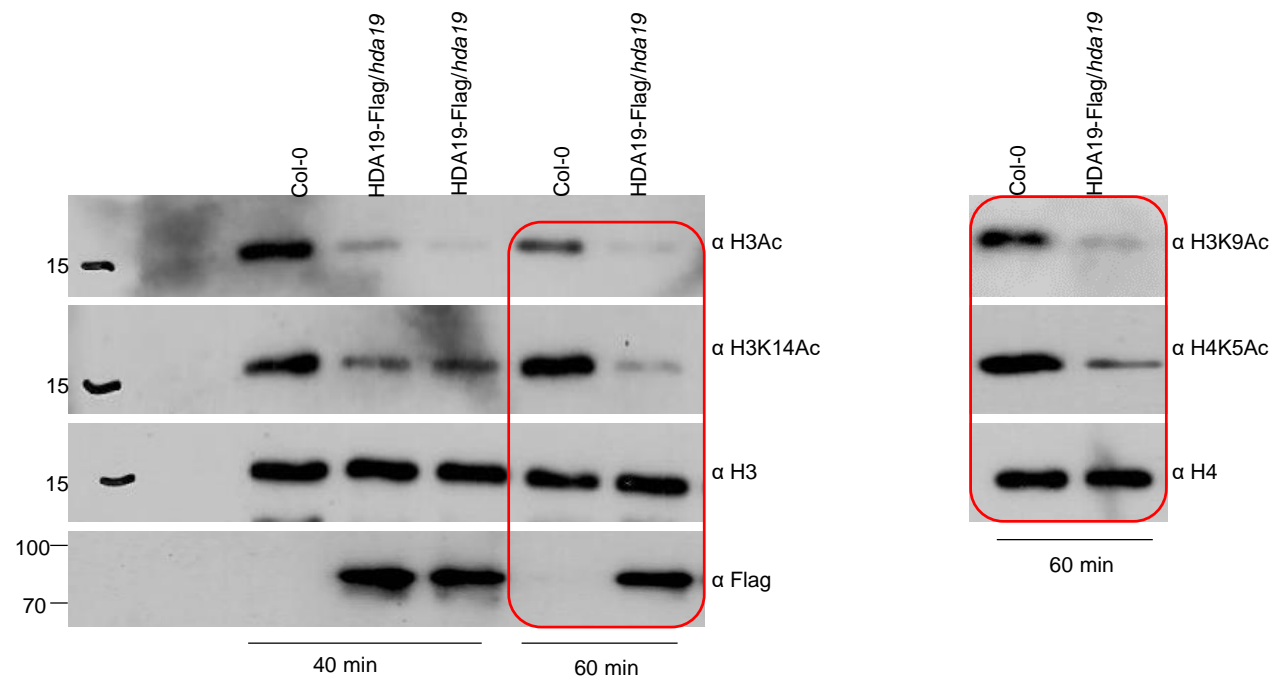

Supplement: Supplementary file 16 — Figure EV4 Source Data [file 44318_2025_445_MOESM16_ESM.zip › Fig EV4A Source Data.pdf]

Fig EV4B

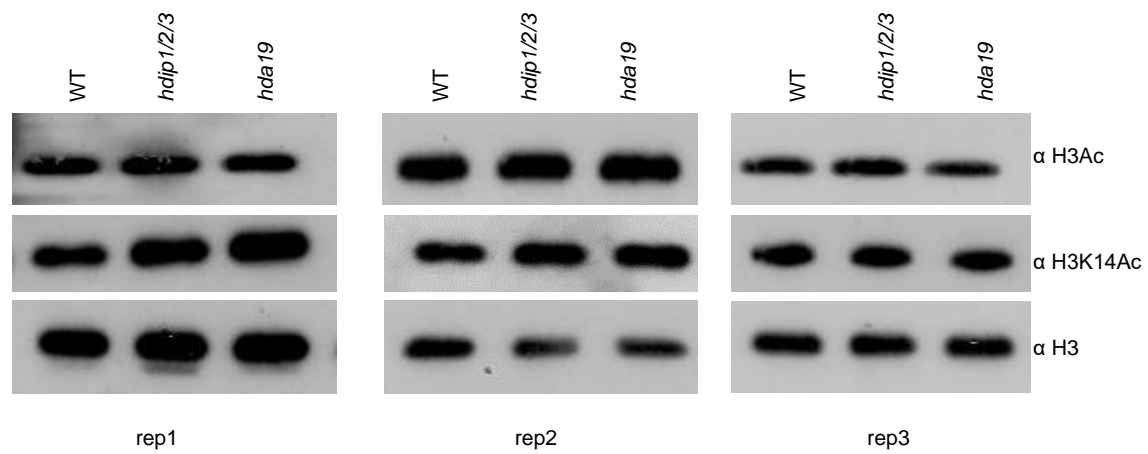

Supplement: Supplementary file 16 — Figure EV4 Source Data [file 44318_2025_445_MOESM16_ESM.zip › Fig EV4B Source Data.pdf]

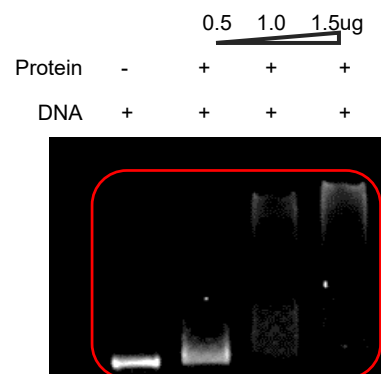

Fig EV5B

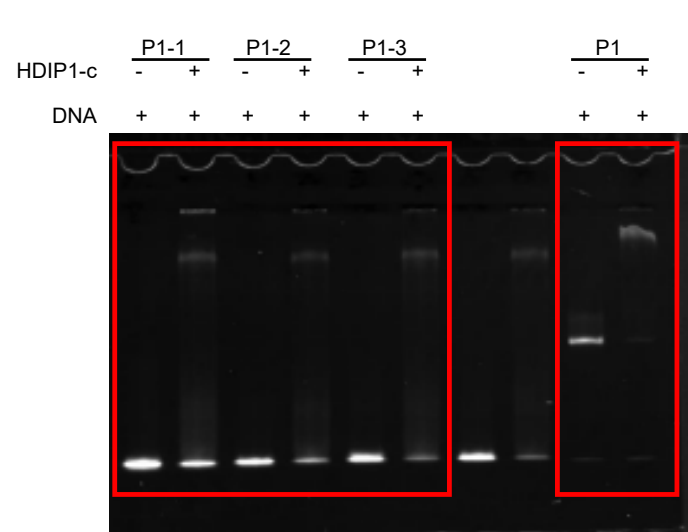

Fig EV5D

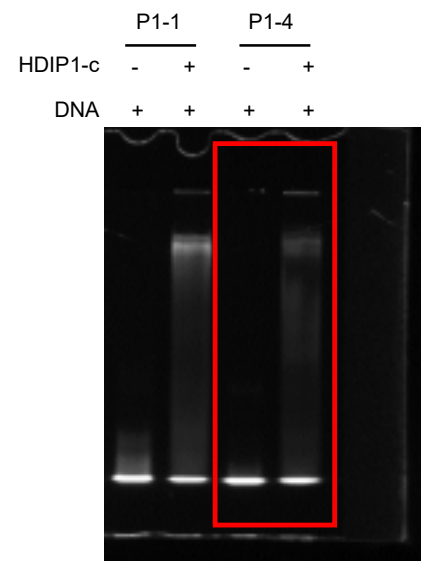

Fig EV5D

Supplement: Supplementary file 17 — Figure EV5 Source Data [file 44318_2025_445_MOESM17_ESM.zip › Fig EV5B 5D Source Data.pdf]

Fig EV5F-H

Fig EV5F

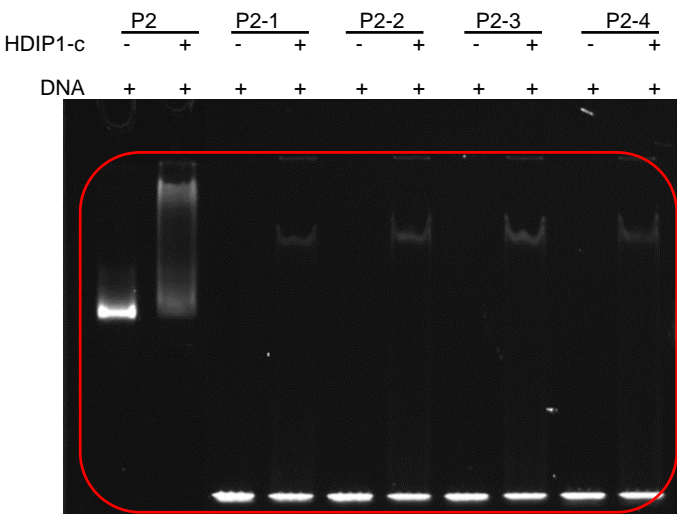

Fig EV5G

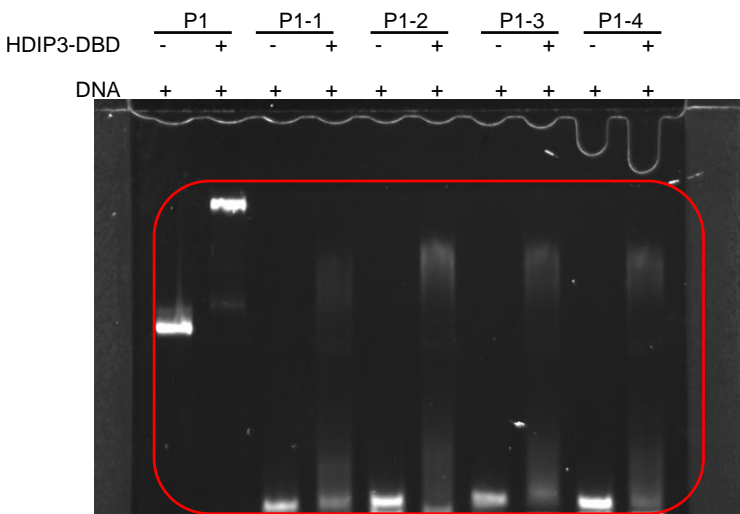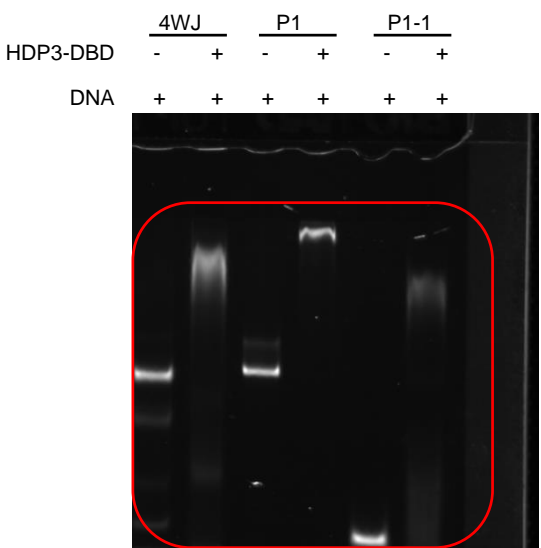

Fig EV5H

Supplement: Supplementary file 17 — Figure EV5 Source Data [file 44318_2025_445_MOESM17_ESM.zip › Fig EV5F 5G 5H Source Data.pdf]

# Appendix Fig S10B, 10C

Appendix Fig S10B

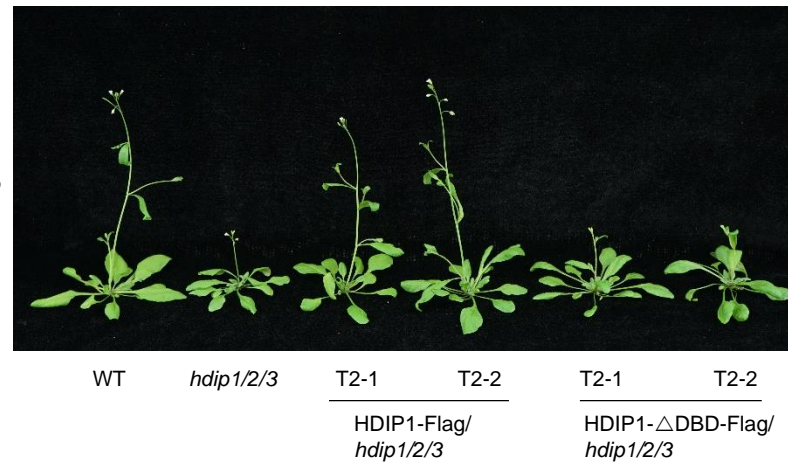

Appendix Fig S10C

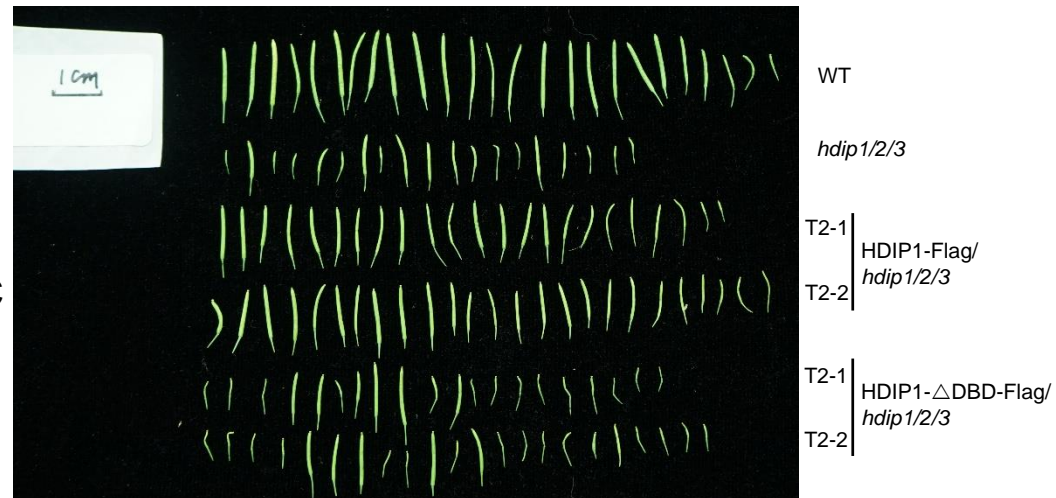

Supplement: Supplementary file 18 — Appendix Figures Source Data [file 44318_2025_445_MOESM18_ESM.zip › Appendix Fig S10B 10C Source Data.pdf]

## Appendix Fig S10A

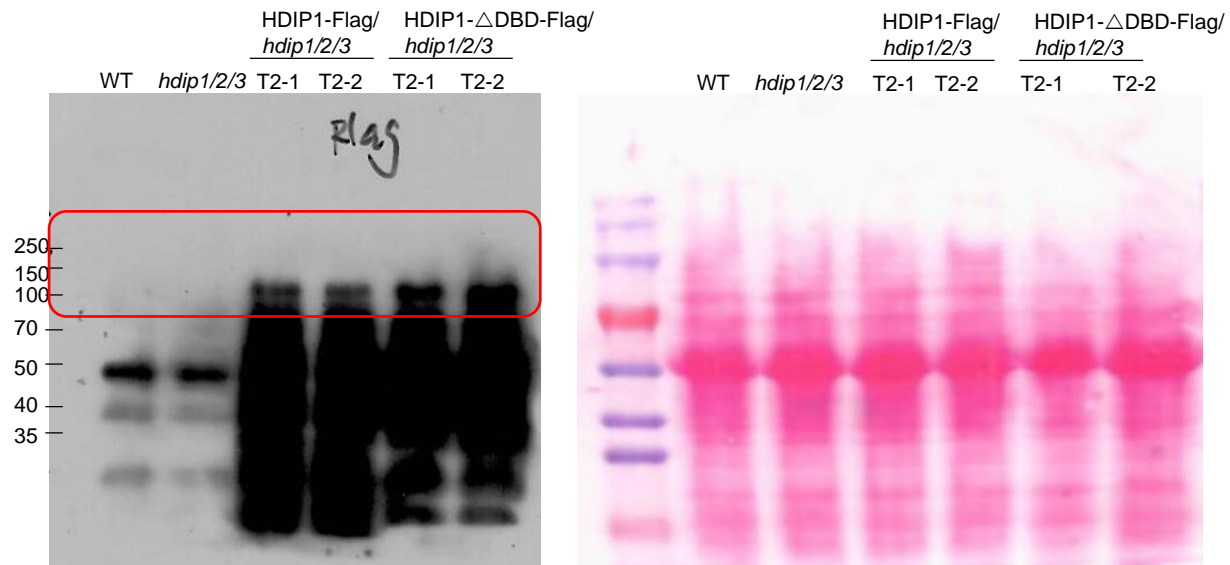

Supplement: Supplementary file 18 — Appendix Figures Source Data [file 44318_2025_445_MOESM18_ESM.zip › Appendix Fig S10A Source Data.pdf]

# Appendix Fig S4A, 4B

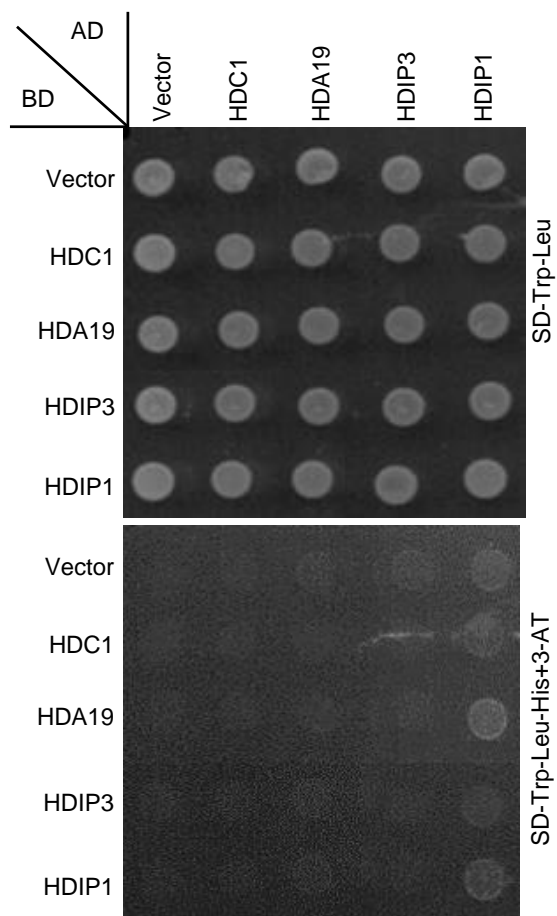

Appendix Fig S4A

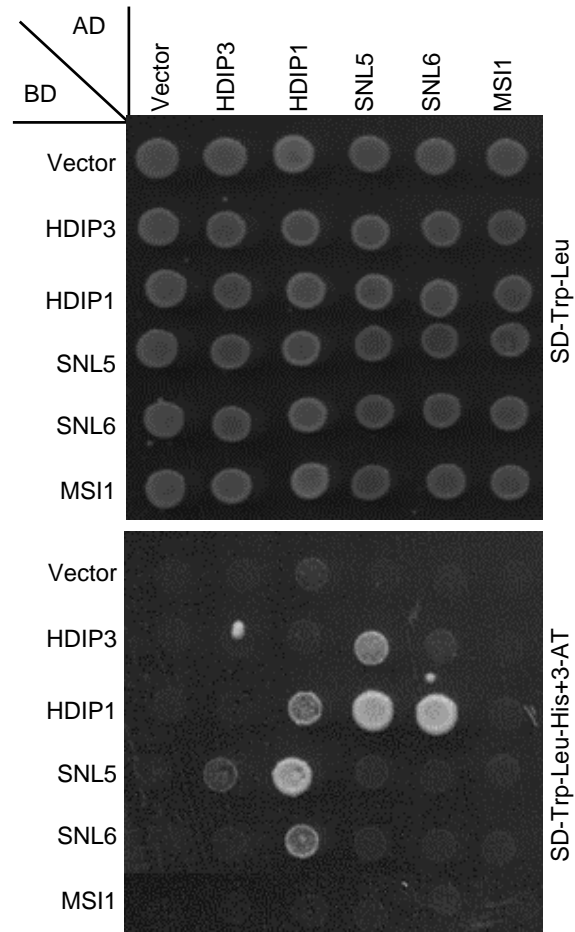

Appendix Fig S4B

Supplement: Supplementary file 18 — Appendix Figures Source Data [file 44318_2025_445_MOESM18_ESM.zip › Appendix Fig S4A 4B Source Data.pdf]

# Appendix Fig S4C, 4D

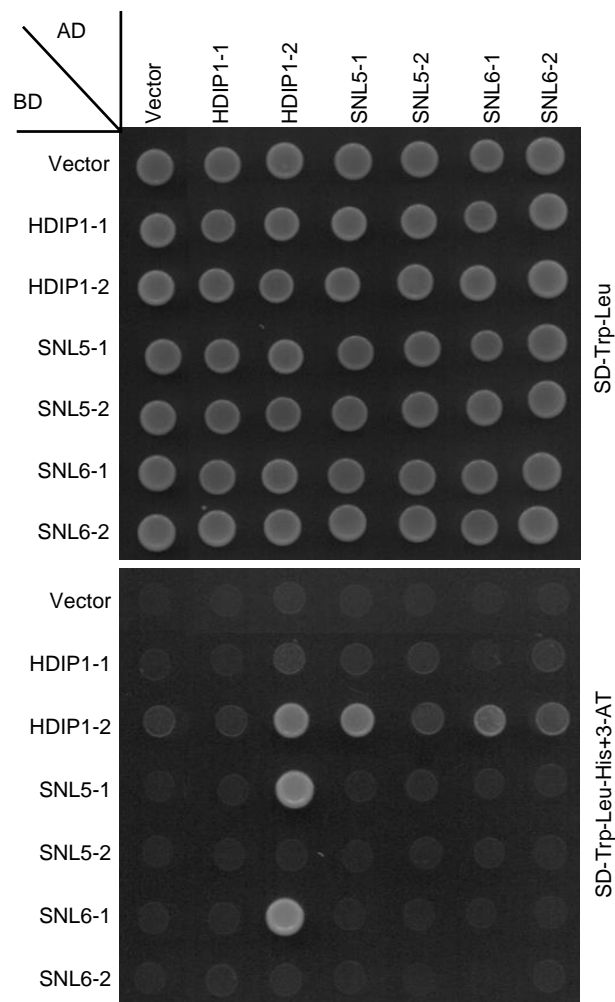

Appendix Fig S4C

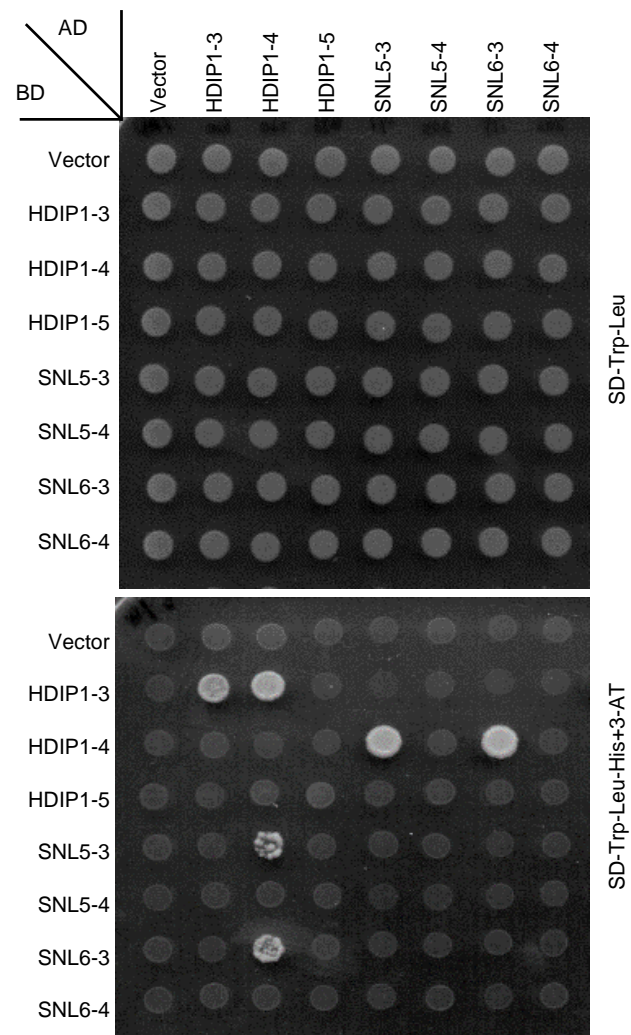

Appendix Fig S4D

Supplement: Supplementary file 18 — Appendix Figures Source Data [file 44318_2025_445_MOESM18_ESM.zip › Appendix Fig S4C 4D Source Data.pdf]

Appendix Fig S5

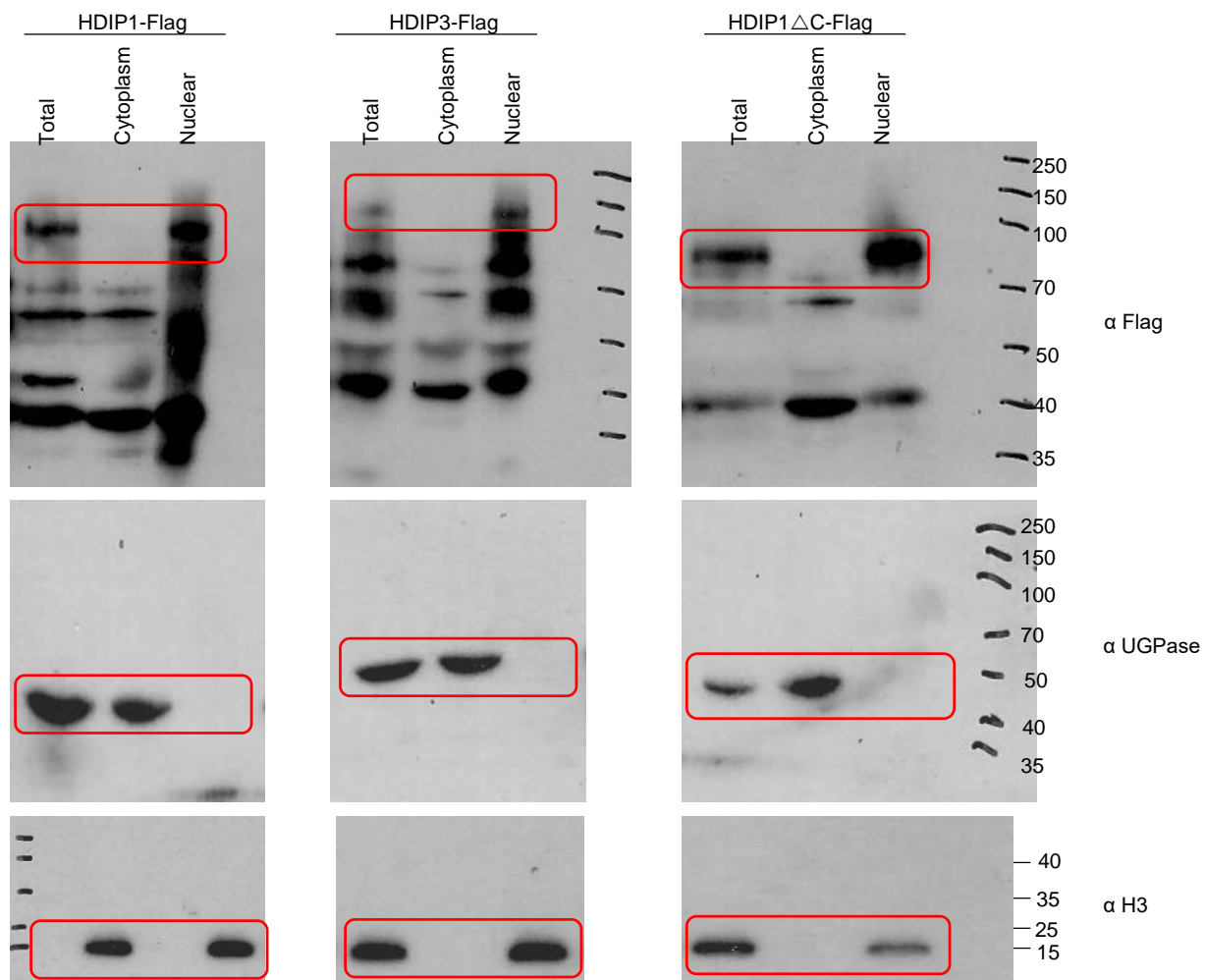

Supplement: Supplementary file 18 — Appendix Figures Source Data [file 44318_2025_445_MOESM18_ESM.zip › Appendix Fig S5 Source Data.pdf]

## Appendix Fig S7A, 7B

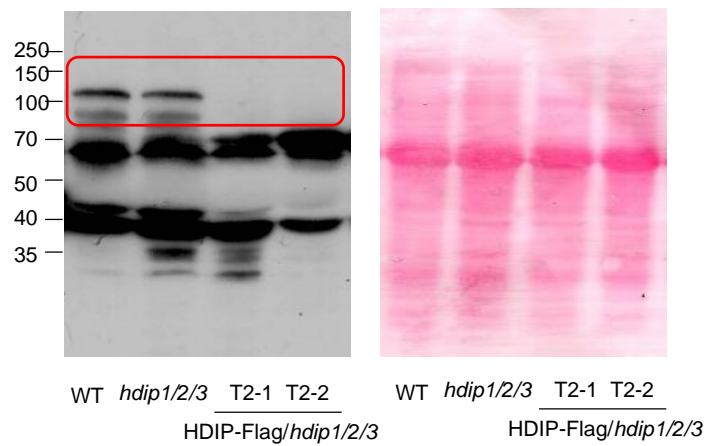

Appendix Fig S7A

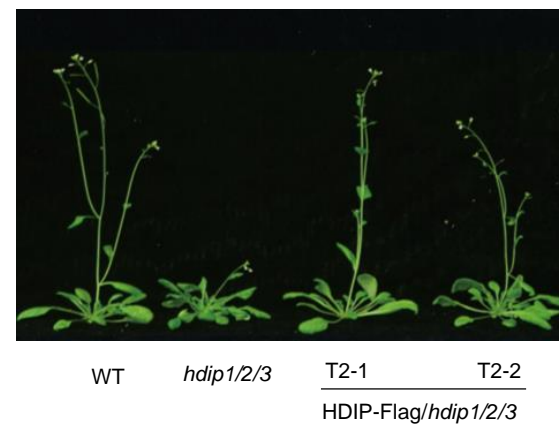

Appendix Fig S7B

Supplement: Supplementary file 18 — Appendix Figures Source Data [file 44318_2025_445_MOESM18_ESM.zip › Appendix Fig S7A Source Data.pdf]

# Appendix Fig S7C, 7H, 7J

Appendix Fig S7C

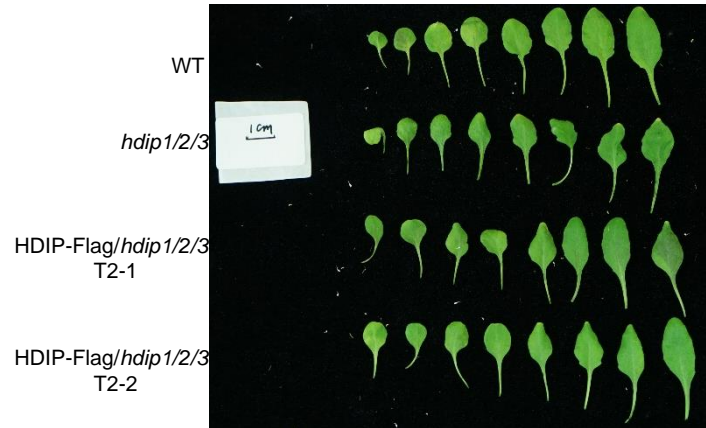

Appendix Fig S7H

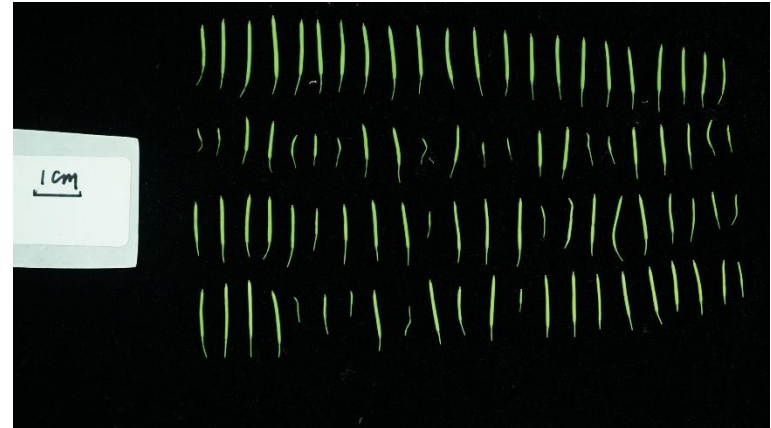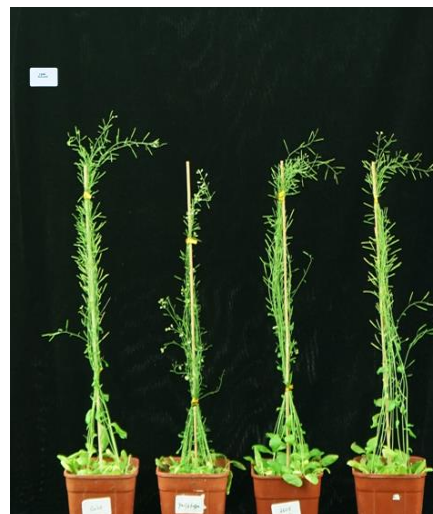

Appendix Fig S7J

WT *hdip1/2/3* T2-1 T2-2  
HDIP-Flag/*hdip1/2/3*

Supplement: Supplementary file 18 — Appendix Figures Source Data [file 44318_2025_445_MOESM18_ESM.zip › Appendix Fig S7C 7C 7J Source Data.pdf]
