# Supplementary material for: Essential angiosperm-specific subunits of HDA19 histone deacetylase complexes in Arabidopsis
Source: EMBO J. 2025 Apr 28;44(12):3521–46. doi: 10.1038/s44318-025-00445-w (PMC12170880; doi:10.1038/s44318-025-00445-w)
Supplement: Supplementary file 19 — Expanded View Figures [file 44318_2025_445_MOESM19_ESM.pdf]

Expanded View Figures

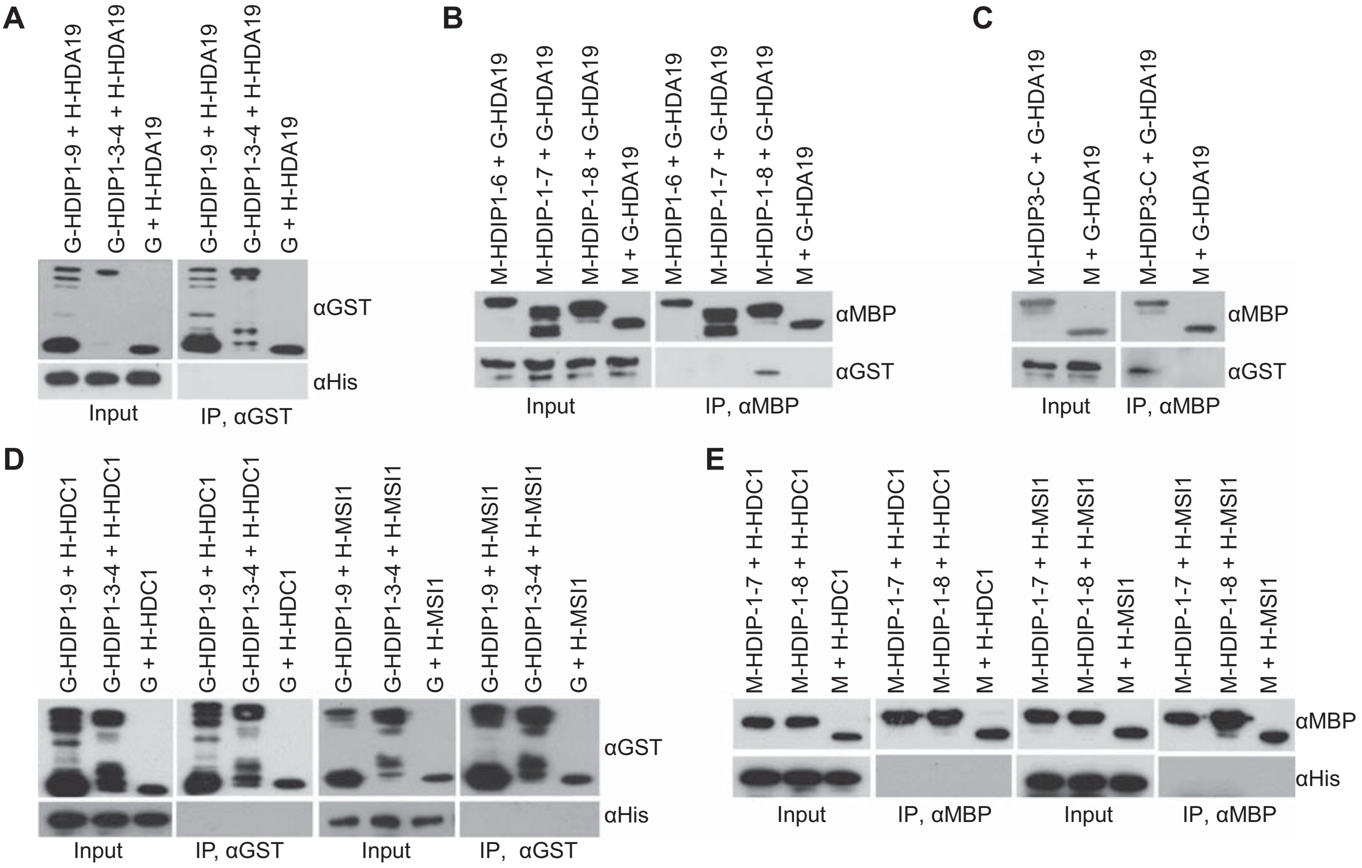

Figure EV1. Interactions of truncated forms of HDIP1/3 with subunits of HDA19-containing histone deacetylase complexes as detected by pull-down assays.

(**A**, **B**) The interaction between HDA19 and truncated forms of HDIP1 was detected by pull-down assays. (**C**) The interaction between HDA19 and HDIP3-C (906–1005 aa) was detected by pull-down assay. (**D**, **E**) The interaction between the truncated forms of HDIP1 and either HDC1 or MSI1 was detected by pull-down assays. G, GST; H, His; M, MBP. The truncated forms of HDIP1 are shown in Fig. 1C. Source data are available online for this figure.

 © The Author(s)

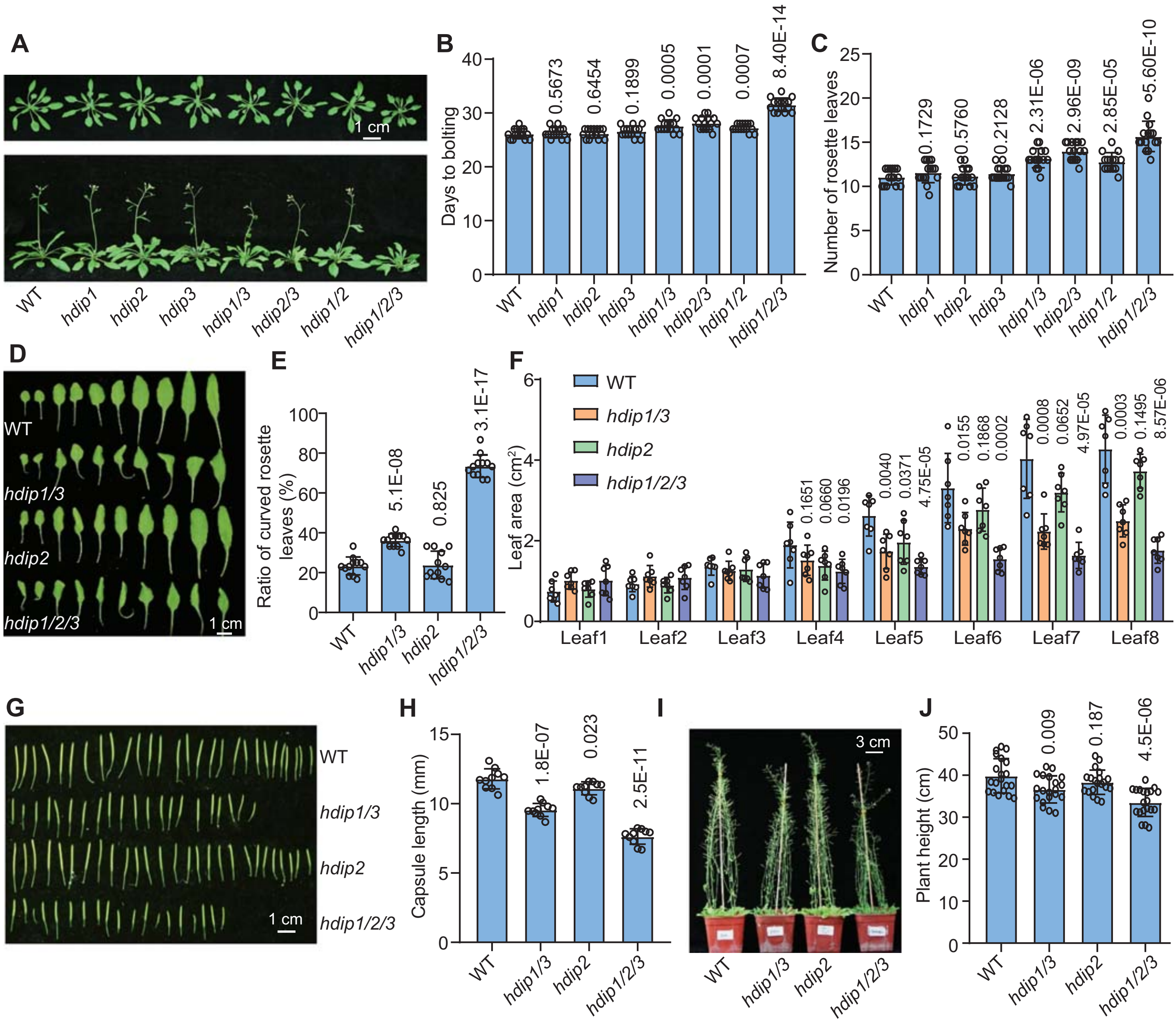

Figure EV2. HDIP1, HDIP2, and HDIP3 redundantly function in the regulation of plant growth and development.

(**A**) Morphological phenotype of flowering time in the wild-type and mutants. (**B**, **C**) Statistical analyses of days to bolting ($n = 18$) (**B**), and the number of rosette leaves ($n = 18$) (**C**). Data are means ± SD. *P* values were determined by two-tailed Student's *t* test. (**D**) Morphological phenotype of rosette leaves from 32-day-old plants. (**E**, **F**) Statistical analysis of the ratio of curved rosette leaves ($n = 12$) (**E**), and leaf area ($n = 8$) (**F**). Data are means ± SD. *P* values were determined by two-tailed Student's *t* test. (**G**) Morphology of siliques in the wild-type and mutants. (**H**) Statistical analysis of the silique length. Data are means ± SD ($n = 10$). *P* values were determined by two-tailed Student's *t* test. (**I**) The plant height phenotype of the wild-type and mutants. (**J**) Statistical analysis of plant height. Data are means ± SD ($n = 20$). *P* values were determined by two-tailed Student's *t* test. Source data are available online for this figure.

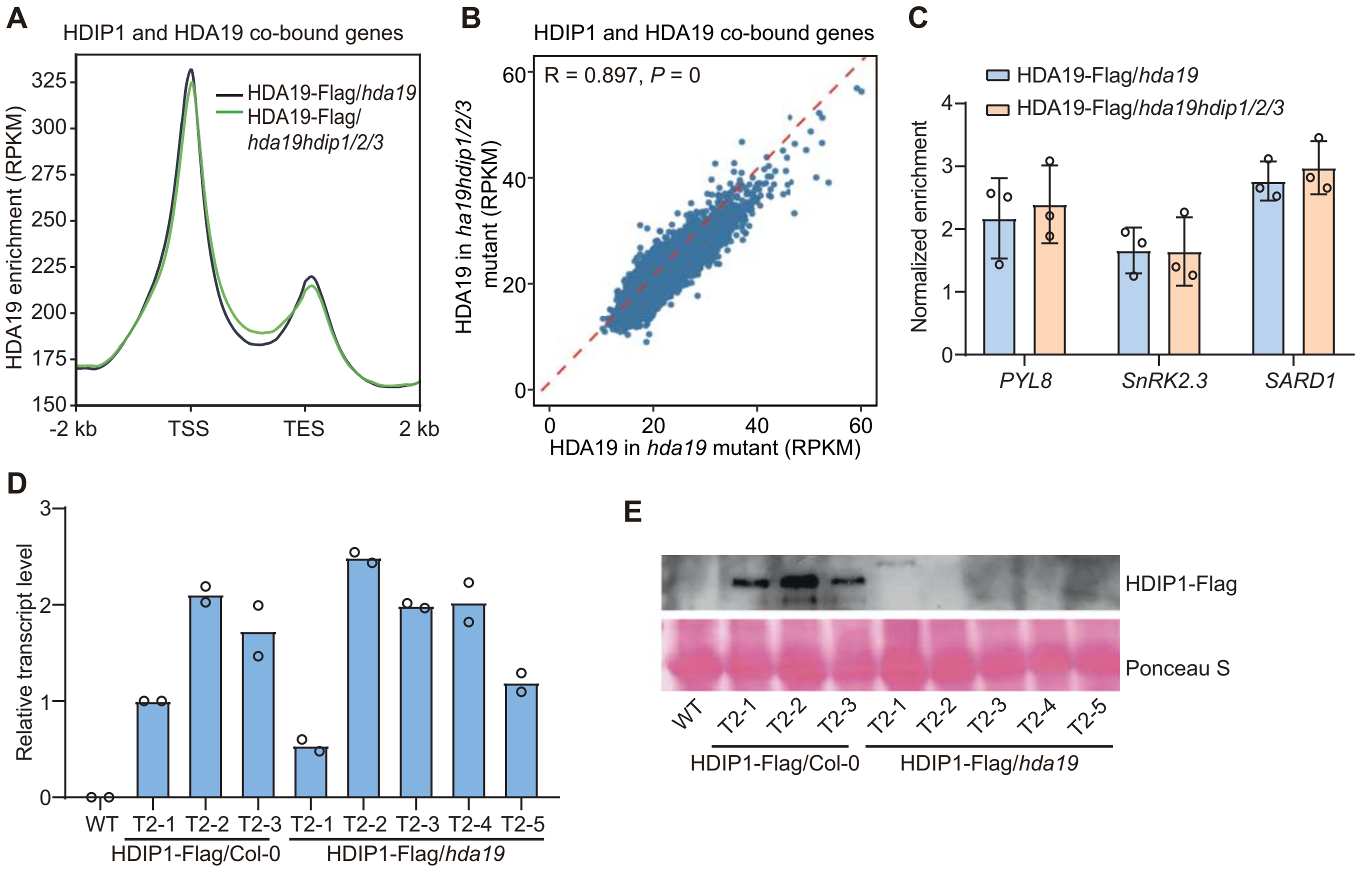

Figure EV3. Determination of the effect of *hdip1/2/3* on the binding of HDA19 to chromatin.

(**A**) Metaplots showing the ChIP-seq signals of HDA19-Flag in *hda19* and *hda19hdip1/2/3* mutant backgrounds at the target genes shared by HDA19 and HDIP1. Data are from two biological replicates. TSS, transcription start site; TES, transcription end site. "-2 kb" and "2 kb" represent the 2-kb regions upstream of TSS and downstream of TES, respectively. (**B**) Scatter plot showing the correlation of HDA19-Flag ChIP-seq signals in *hda19* and *hda19hdip1/2/3* mutant backgrounds at the target genes shared by HDA19 and HDIP1. Data are based on two biological replicates. The Pearson correlation coefficient (R) and the associated significance (*P* values) are shown. *P* values were determined by two-sided Pearson correlation test. (**C**) The enrichment of HDA19-Flag at the *PYL8*, *SnRK2.3* and *SARD1* loci determined by ChIP-qPCR in *hda19* and *hda19hdip1/2/3* mutant backgrounds. The chromatin from MCF7 cells (human) with Flag-ESR1 was added to the target chromatin and used as a spike-in control. *GREB1* represents the positive locus of the spike-in genome. Bar are means of three independent biological replicates ± SD. (**D**) The expression levels of *HDIP1-Flag* in the wild-type and *hda19* mutant backgrounds as determined by quantitative RT-PCR. Data are from two biological replicates. (**E**) Determination of the expression of the *HDIP1-Flag* in the wild-type and *hda19* mutant backgrounds by western blot analysis. Ponceau S-stained ribosome proteins are shown as a loading control. Source data are available online for this figure.

© The Author(s)

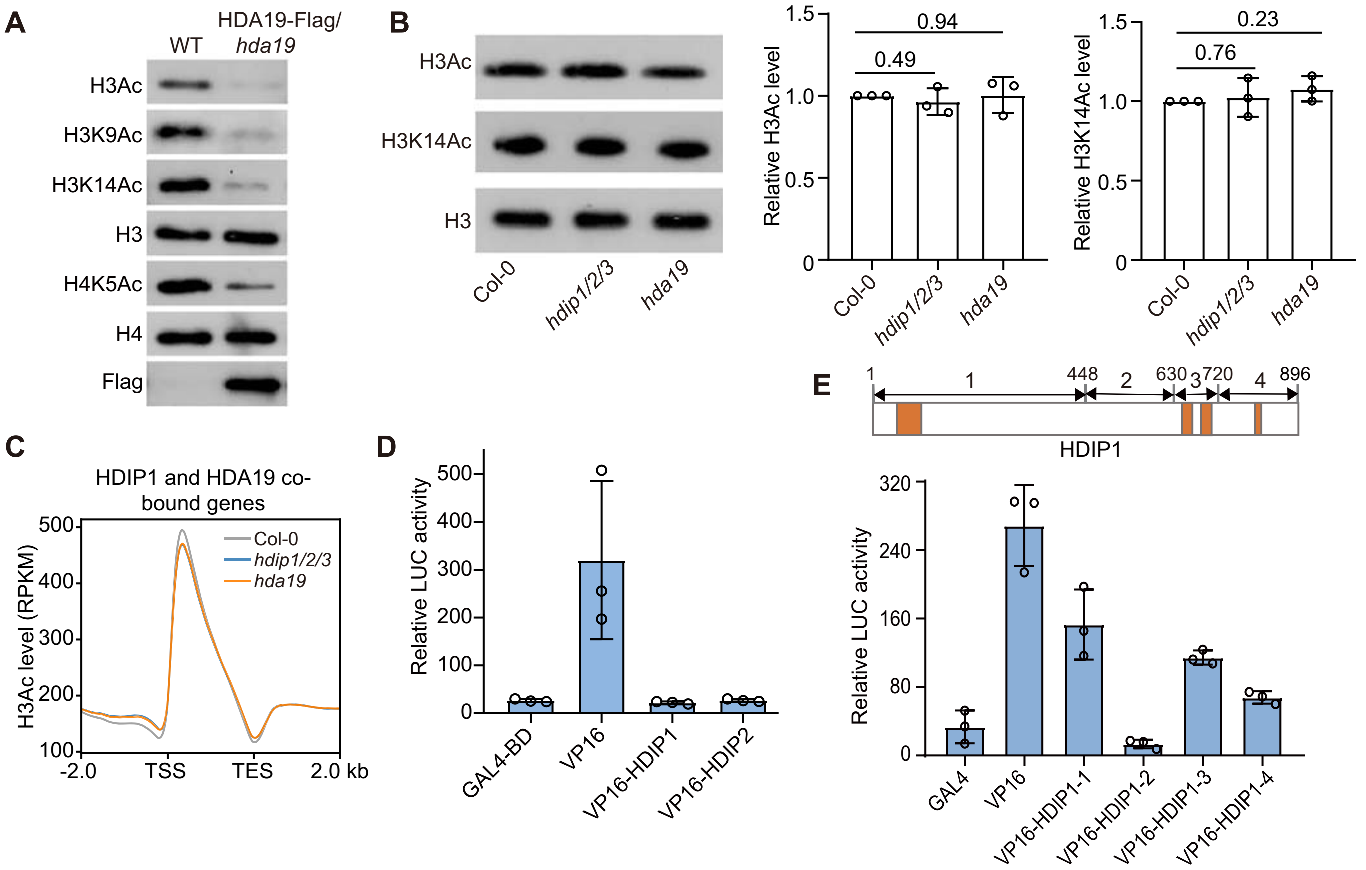

Figure EV4. The roles of HDA19 and HDIP1/2/3 in histone deacetylation and transcriptional repression.

(**A**) Determination of the histone deacetylase activity of the HDA19-containing complex. The HDA19-containing complex was purified from *HDA19-Flag* transgenic plants, and incubated with free histone substrates from calf thymus for the histone deacetylation assay. Immunoblots signals were detected by anti-H3Ac, anti-H3K9Ac, anti-H3K14Ac, and H4K5Ac antibodies. (**B**) Detection of the effect of *hda19* and *hdip1/2/3* mutants on H3 acetylation by western blot analysis. Quantifications of the H3Ac and H3K14Ac are shown in bar graphs. *P* values were determined by two-tailed Student's *t* test. (**C**) Metaplots showing the average distribution of H3Ac at HDIP1 and HDA19 shared target genes in Col-0, *hdip1/2/3*, and *hda19* mutants. (**D**) The transcriptional repression capacity of HDIP1 and HDIP2 as determined by the LUC reporter assay. Values are means ± SD of three biological replicates. (**E**) Determination of transcriptional repression ability of truncated HDIP1 by the LUC reporter assay. Diagrams represent truncated versions of the HDIP1 protein (upper). Truncated HDIP1-1 (1–448 aa), HDIP1-2 (449–630 aa), HDIP1-3 (631–720 aa), and HDIP1-4 (721–896 aa) were fused with the VP16 activation domain driven by the *CaMV 35S* promoter. Values are means ± SD of three independent biological replicates. Source data are available online for this figure.

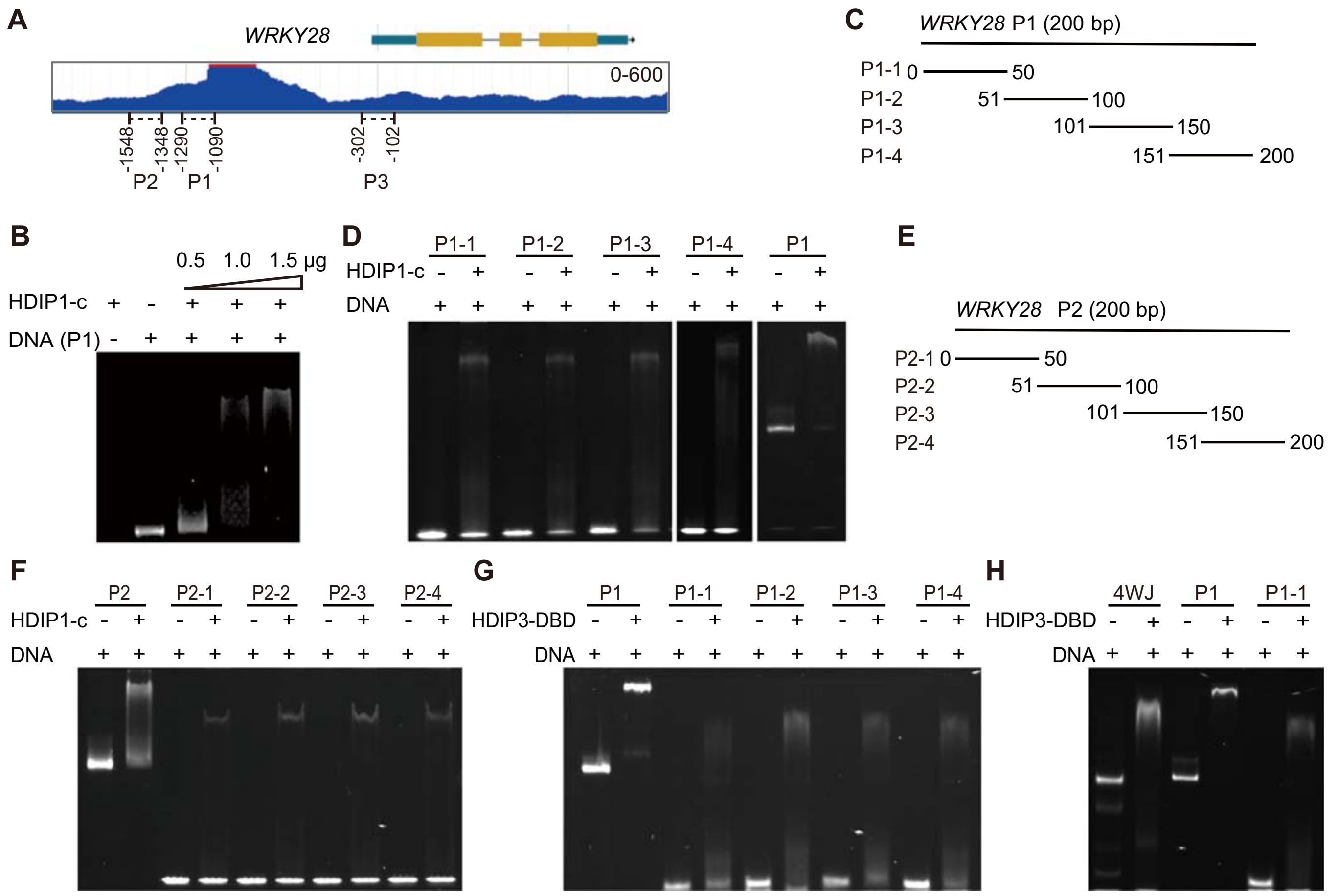

Figure EV5. Determination of the binding ability of HDIP1 and HDIP3 with various DNA probes by EMSA.

(**A**) The HDIP1 ChIP-seq signal across the *WRKY28* gene and the locations of the 200-bp P1, P2, and P3 DNA probes. (**B**) The binding of HDIP1 to the 200-bp double-stranded probe P1 as determined by EMSA. Increasing amounts of the HDIP1-c protein were used in the binding reaction mixture. (**C**) Schematic representations of the complete 200-bp DNA probe P1 and its truncated 50-bp versions: P1-1, P1-2, P1-3, and P1-4. (**D**) The binding of HDIP1-c to the 200-bp DNA probe P1 and its truncated 50-bp derivatives as determined by EMSA. (**E**) Schematic representations of the 200-bp DNA probe P2 and its truncated 50-bp versions: P2-1, P2-2, P2-3, and P2-4. (**F**) The binding of HDIP1-c to the 200-bp DNA probe P2 and its truncated 50-bp derivatives as determined by EMSA. (**G**) The binding of HDIP3-DBD (806–930 aa) to the 200-bp DNA probe P1 and its truncated 50-bp derivatives as determined by EMSA. (**H**) Determination of the binding ability of HDIP3-DBD with 4WJ DNA by EMSA. The 50-bp P1-1 probe and the 200-bp P1 probe were used as controls. Source data are available online for this figure.

 © The Author(s)
